# Disease-guided targeted protein degradation and clearance: matching strategies to pathological protein states

**DOI:** 10.3389/fmed.2026.1967704

**Published:** 2026-10-15

**Authors:** Tianyu Wang, Chenbo Ji, Tianyu Zhao

**Affiliations:** 1 Department of Pharmacy, The First Affiliated Hospital of Harbin Medical University, Harbin, Heilongjiang, China; 2 Jiangsu Key Laboratory of Drug Design and Optimization, Department of Medicinal Chemistry, School of Pharmacy, China Pharmaceutical University, Nanjing, Jiangsu, China

**Keywords:** lysosome-targeted clearance, molecular glues, pathological protein states, patient stratification, precision therapy, proteolysis-targeting chimeras, selective autophagy, targeted protein degradation

## Abstract

Targeted protein degradation (TPD) and related targeted-clearance strategies expand therapeutic opportunities by eliminating disease-associated proteins and, in selected settings, damaged organelles. Rather than organizing the field by degrader modality or disease lineage, this review uses the dominant pathological state of the disease-causing species as the unit of analysis. We classify targets along three higher-order dimensions—abundance/functional, physical, and spatial/compartmental state—and resolve them into five operational categories: elevated abundance or aberrant dependency; persistent activation, gain-of-function alterations, or resistance mutations; misfolding or pathological conformations; oligomerization, fibril formation, or insoluble aggregation; and mislocalization, abnormal cell-surface presentation, or organelle damage. Across molecular glues, heterobifunctional degraders, endocytic–lysosomal systems, and autophagy-based approaches, we examine how pathological state constrains recognition chemistry, molecular accessibility, clearance-route compatibility, tissue exposure, and pharmacodynamic assessment. The 2026 approval of vepdegestrant represents an important milestone in the clinical translation of heterobifunctional protein degradation and highlights the therapeutic potential of targeted degradation strategies. We propose a pathological-state–clearance pathway–biomarker framework to guide modality selection, medicinal-chemistry optimization, mechanistic validation, and patient stratification.

## Introduction

## 1 Proteostasis disruption and a disease-guided framework for targeted protein degradation (TPD)

### 1.1 Disease-associated protein abnormalities

The pathological effects of disease-associated proteins depend not only on catalytic activity or canonical function but also on protein abundance, conformational state, post-translational modifications, interaction networks, and subcellular localization (1–5). The same protein may serve distinct roles across tissues, cell types, and subcellular environments. Its pathogenic effects and pharmacological dependencies are therefore strongly context-dependent (5–7). In cancer, neurodegenerative disorders, immune-mediated inflammatory diseases, and metabolic disorders, disrupted proteostasis can drive abnormal proliferation, neurotoxicity, chronic inflammation, and metabolic imbalance, respectively (8–11).

From the perspectives of pharmacology and clearance mechanisms, disease-associated protein abnormalities can be grouped into three interrelated higher-order dimensions. The first comprises changes in abundance or functional state, including elevated expression, impaired turnover, persistent activation, gain-of-function alterations, and resistance mutations selected during treatment (3, 12–14). The second involves changes in physical state, such as conformational instability, misfolding, oligomerization, fibril formation, and insoluble aggregation (15–18). The third encompasses abnormal spatial states of proteins or their associated structures, including nucleocytoplasmic mislocalization, aberrant cell-surface expression, extracellular accumulation, and organelle damage (3, 5, 19–21). These abnormalities may coexist within the same protein, yet they differ in their effects on ligand recognition, clearance pathways, and the therapeutic window (22–25).

Traditional organization of TPD development according to individual technologies, such as PROTACs, molecular glues, LYTACs, or AUTACs, provides a useful mechanistic description but may not fully capture the therapeutic decision process. A shared pathological state of disease-associated species can arise from distinct molecular mechanisms across diseases, whereas the same clearance strategy may be applicable to unrelated disease contexts when the target shares comparable biological constraints.

To translate these three higher-order dimensions into an operational classification for selecting clearance modalities, we further group disease-associated targets into five categories. These comprise elevated protein abundance and aberrant dependency; persistent activation, gain-of-function alterations, and resistance mutations; protein misfolding and pathological conformations; oligomerization, fibril formation, and insoluble aggregation; and spatial mislocalization, abnormal cell-surface presentation, and organelle damage. These five categories are operational rather than mutually exclusive. A single protein may occupy multiple pathological states simultaneously or sequentially during disease progression (3, 5, 18). For classification, we apply a hierarchical rule: first, identify the disease-causing protein species directly targeted by the intervention; second, determine which abnormal feature most strongly constrains molecular recognition and physical accessibility; and third, assign the target to the category that most directly determines clearance-route selection and the relevant pharmacodynamic readout (22, 26, 27). Other abnormalities are treated as secondary modifiers rather than grounds for duplicate classification. Thus, the framework classifies the therapeutically targeted pathological species rather than the protein identity itself. The resulting pathological-state framework and its relationship to compatible clearance routes are summarized in Figure 1.

**Figure 1:**
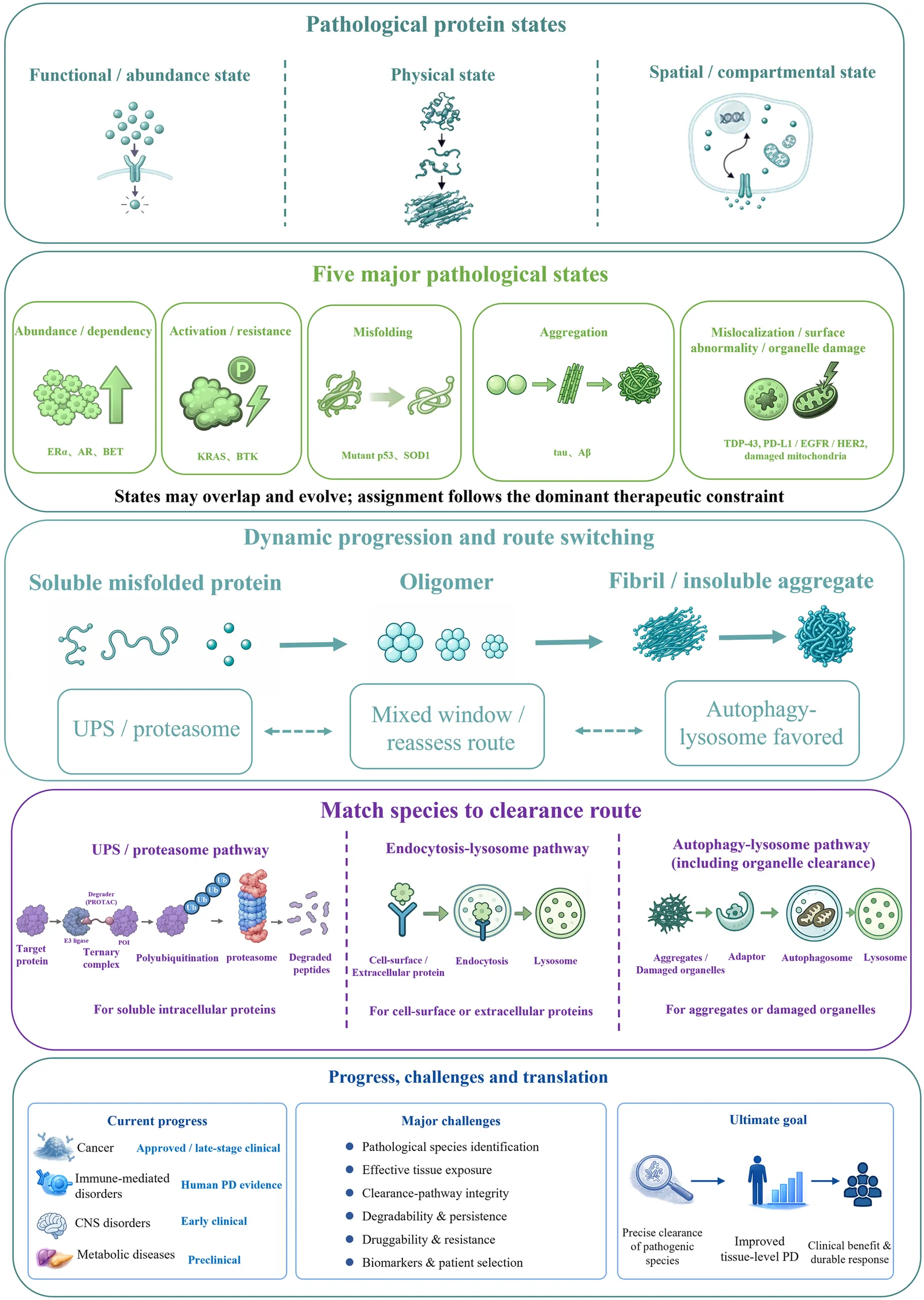
Disease-guided framework linking pathological protein states to targeted clearance and therapeutic translation. Disease-associated protein abnormalities are organized across three higher-order dimensions-functional or abundance, physical, and spatial/compartmental states-into five major, non-mutually exclusive pathological categories. These states may overlap and evolve during disease progression and are matched to compatible clearance routes according to the dominant therapeutic constraint, with soluble intracellular proteins generally favoring the ubiquitin-proteasome system, cell-surface or extracellular proteins favoring endocytosis–lysosome pathways, and aggregates or damaged organelles favoring autophagy-lysosome pathways. This framework links pathological-state classification with clearance-route selection and highlights current progress, key translational challenges, and the ultimate goal of achieving precise, effective, and durable therapeutic responses.

### 1.2 Pharmacological evolution of TPD and expansion of its clearance scope

Conventional protein-targeted drugs alter target activity by occupying, occluding, or modulating functional sites (28). Small molecules typically bind orthosteric or allosteric pockets, whereas monoclonal antibodies recognize extracellular or cell-surface epitopes (29). Tamoxifen, trastuzumab, and imatinib established the clinical value of nuclear receptor modulation, antibody-based targeting, and kinase inhibition, respectively (30–32). These strategies now follow well-established paths for drug design and clinical development (28–32). However, the effects of many reversible inhibitors remain constrained by drug exposure, target occupancy, and binding duration (33). Functional inhibition alone is often insufficient to eliminate disease dependency when target amplification increases protein abundance or bypass signaling, binding-site mutations reduce drug affinity, or non-catalytic functions sustain the pathological process (12, 34–36).

In 2001, Sakamoto and colleagues developed PROTAC-1, which recruited SCF^β-TRCP^ to induce the ubiquitination and degradation of methionine aminopeptidase 2 (MetAP-2), providing the first proof of concept for proteolysis-targeting chimeras (PROTACs) (37). In 2004, Schneekloth and colleagues used a von Hippel–Lindau (VHL)-recognition peptide to achieve degradation of a fusion protein in cultured cells (38). Because early PROTACs relied on peptidic E3 ligase–binding moieties, they had limited cell permeability and drug-like properties and therefore served primarily as proof-of-concept tools (37–39).

In 2014, lenalidomide was shown to induce the degradation of IKZF1 and IKZF3 by reshaping substrate recognition by cereblon (CRBN) (40, 41). The following year, fully small-molecule PROTACs enabled efficient target degradation *in vivo* (42, 43). These advances moved TPD from proof of concept toward a pharmacological modality amenable to systematic design. Molecular glues redirect E3 ligase substrate recognition, whereas heterobifunctional degraders induce proximity between a target protein and an E3 ligase (42–44). Subsequent efforts increasingly focused on the systematic optimization of potency, selectivity, and pharmacokinetic properties (44).

Structural and biophysical studies further showed that PROTAC activity is governed by the ternary complex as a whole, rather than by the simple combination of the binding affinities of its two ligands. Conformational complementarity, cooperativity, and dissociation kinetics jointly determine degradation efficiency and selectivity (45–47). Degrader optimization therefore shifted from maximizing binary affinity to controlling the induced protein–protein interface and its dynamic behavior.

Around 2019, targeted clearance began to extend beyond the ubiquitin–proteasome system (UPS) into the autophagy–lysosomal and endocytic–lysosomal networks. Autophagy-targeting chimeras (AUTACs) and autophagosome-tethering compounds (ATTECs) can direct pathological proteins or damaged organelles to selective autophagy, whereas lysosome-targeting chimeras (LYTACs) use receptor-mediated endocytosis to clear cell-surface and extracellular proteins (26, 27, 48). These platforms broaden the physical scale and spatial range of substrates amenable to clearance. Their efficacy, however, remains constrained by target accessibility, the capacity of the clearance machinery, and tissue-specific expression patterns (26, 27, 48, 49).

In clinical translation, the oral estrogen receptor (ER) degrader vepdegestrant significantly prolonged progression-free survival in patients with ESR1-mutant advanced breast cancer in the phase III VERITAC-2 trial and received approval from the US Food and Drug Administration (FDA) on May 1, 2026 (50, 51). The KRAS G12D degrader setidegrasib has shown preliminary antitumor activity in humans and has entered phase III clinical development (52, 53). These advances mark the transition of TPD from a tool for protein modulation to an approved therapeutic modality with candidates in late-stage clinical development. Its clinical value, however, continues to depend on disease dependency, patient stratification, tissue exposure, and the therapeutic window. The pharmacological evolution and expansion of targeted clearance are summarized in Figure 2.

**Figure 2:**
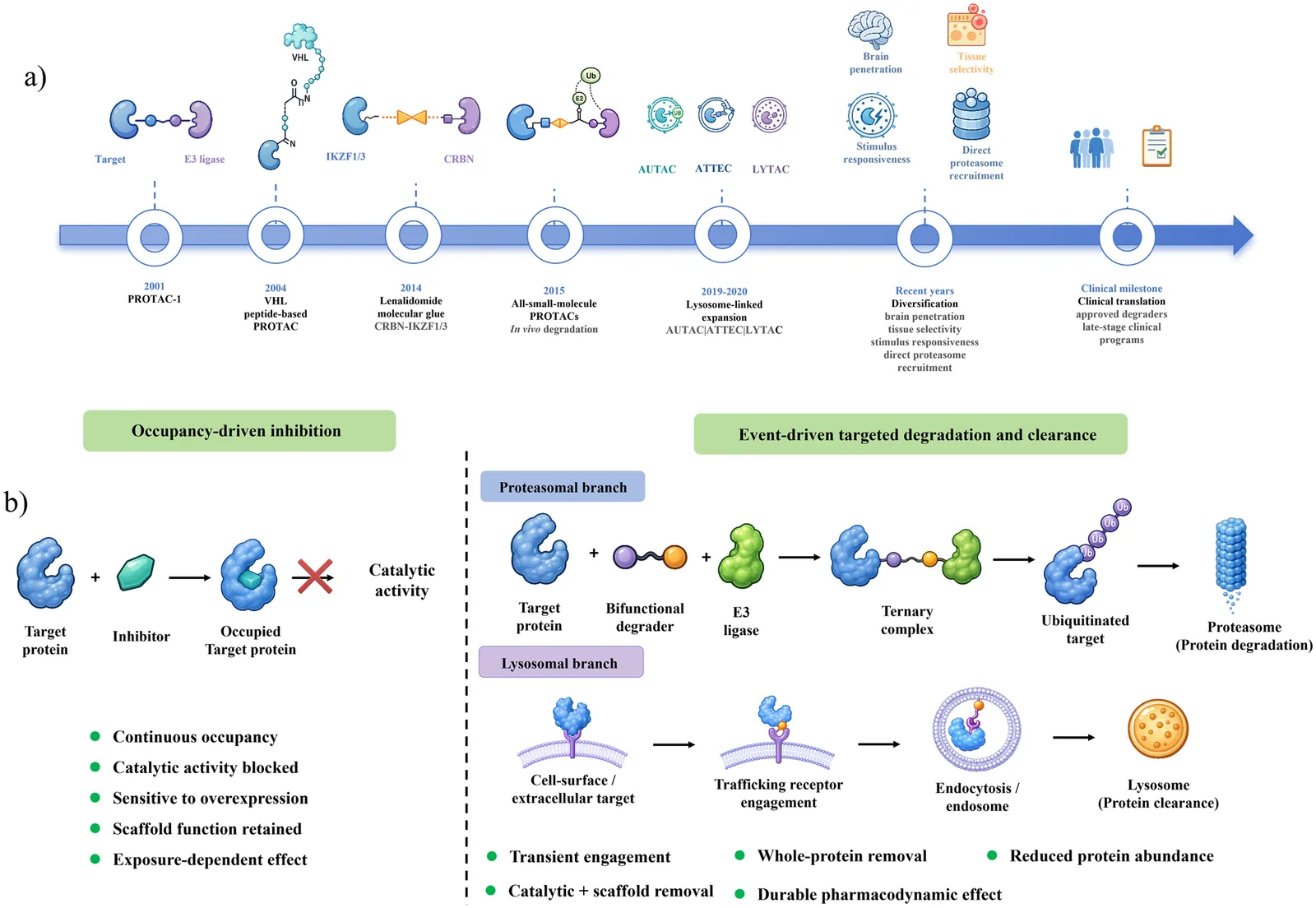
Evolution and expansion of TPD. **(a)** Key milestones in the evolution of TPD, from occupancy-driven pharmacology and early PROTACs to molecular glues, small-molecule degraders, expanded lysosomal clearance strategies, and clinical translation. **(b)** Conceptual transition from occupancy-driven pharmacology to event-driven targeted degradation and clearance. Proteasome-directed degraders promote E3-ligase-dependent ubiquitination of intracellular proteins, whereas cell-surface or extracellular targets can be redirected through receptor-mediated endocytosis to lysosomes, and selected intracellular aggregates or damaged organelles can be cleared through autophagy–lysosomal pathways.

### 1.3 Functional inhibition and targeted degradation: complementary pharmacological strategies

Functional inhibition and targeted degradation represent two complementary pharmacological strategies. Occupancy-driven drugs require sustained target engagement to modulate protein activity and are generally suited to targets with tractable functional sites whose pathological effects are driven primarily by blockable functional outputs (33, 54). Targeted degradation instead uses reduced protein abundance as the principal pharmacodynamic event, thereby suppressing catalytic activity, scaffolding roles, and other non-catalytic functions (42, 54). Degradation may therefore offer an advantage when disease dependency is sustained by the continued presence, elevated abundance, or non-catalytic functions of the target protein (34, 36, 55).

The two strategies are not mutually exclusive; the choice should be guided by the target's dominant pathological state, the nature of disease dependency, and the pharmacological effect that can be achieved. Whether degradation provides greater therapeutic value than functional inhibition depends on the degree of target dependency in diseased tissue and is further constrained by protein resynthesis, tissue exposure, availability of the clearance machinery, and tolerance in normal tissues (56, 57). When selective inhibition is sufficient to control the critical function and offers a clear therapeutic window, further reducing protein abundance may confer little additional benefit. By contrast, TPD may provide distinct value when disease depends on both catalytic and non-catalytic functions (36, 55), resistance mutations weaken inhibitor activity, or sustained reduction of the pathological protein burden is required (57).

This review adopts a broad framework of targeted protein clearance that encompasses molecular glues, heterobifunctional proteasome-directed degraders, endocytosis- or autophagy-mediated clearance strategies, and organelle-targeted clearance closely linked to the disposal of aberrant proteins (26, 27, 44, 48). Representative evidence was drawn primarily from published studies that established the mechanism of action and reported quantitative protein depletion, *in vivo* pharmacodynamic effects, or clinical outcomes. The half-maximal degradation concentration (DC_50_) and maximum degradation (D_max_) vary with cellular context, treatment duration, dosing regimen, and assay method (57–59). We therefore do not rank values across distinct experimental systems and compare relative differences only under similar experimental conditions.

Rather than using degrader modality, cellular clearance platform, or disease lineage as the primary organizing framework, this review treats the dominant pathological state of disease-associated proteins as the unit of analysis. This approach considers pathological state as the upstream determinant that defines molecular accessibility, recognition selectivity, clearance-route compatibility, and measurable pharmacodynamic outcomes. By comparing proteins with similar pathological alterations across diseases and targets, we identify shared factors that govern target accessibility, compatibility with clearance machinery, selectivity for pathological species, and the therapeutic window (44, 56, 57). We also define common bottlenecks in clearance-strategy selection, mechanistic validation, and clinical translation for each pathological state. Building on these comparisons, we derive transferable design principles and evaluation strategies to guide clearance-pathway selection, validation-system development, and patient stratification for proteins that share similar pathological states but currently lack sufficient TPD evidence. The five pathological states, representative targets, preferred clearance routes, and corresponding validation requirements are summarized in Table 1.

**Table 1:** Disease-guided classification of pathological protein states and matched preferred clearance strategies.

Higher-order dimension	Pathological state	Representative targets	Clearance route	Key design requirement	Biomarker/validation
Abundance/function	Elevated abundance/dependency	ERα, AR, BET, SMARCA2, IKZF1/3, BCL-XL	UPS	Full-length depletion with therapeutic window	Target dependency; tissue depletion
Abundance/function	Activation/gain of function/resistance	KRAS, BTK, EGFR, IRAK4, RIPK1/2, LRRK2	UPS	Mutation coverage; remove intact protein functions	Mutation status; pathway suppression
Physical state	Misfolding/pathological conformation	p53, SOD1	UPS/lysosome	Conformation selectivity; spare normal protein	Pathological conformer; normal-protein preservation
Physical state	Oligomers/fibrils/aggregates	Aβ, tau, α-synuclein	Autophagy/endocytosis	Match clearance capacity to aggregate scale	Aggregate burden; seeding activity
Spatial state	Mislocalization/surface abnormality/organelle damage	TDP-43, PD-L1/EGFR/HER2, damaged mitochondria	Compartment-dependent	Spatially selective clearance	Compartment-specific pharmacodynamics

Pathological states are non-mutually exclusive. Assignment reflects the dominant disease-causing species and the feature that most strongly determines target recognition, physical accessibility, clearance-route selection, and pharmacodynamic assessment; coexisting abnormalities are treated as secondary modifiers.

Aβ, amyloid-β; AR, androgen receptor; BET, bromodomain and extraterminal; EGFR, epidermal growth factor receptor; ERα, estrogen receptor alpha; HER2, human epidermal growth factor receptor 2; PD, pharmacodynamics; PD-L1, programmed death-ligand 1; TDP-43, TAR DNA-binding protein 43; UPS, ubiquitin–proteasome system.

## 2 Pathological protein states and targeted clearance

The following sections are organized around five operational pathological states rather than mutually exclusive protein classes. For targets with multiple abnormalities, assignment is based on the pathological species directly addressed by the intervention and the feature that most strongly determines ligand accessibility, substrate scale, clearance-route compatibility, and pharmacodynamic assessment. Other coexisting abnormalities are treated as secondary modifiers. Accordingly, the same protein may enter different categories in different disease or therapeutic contexts. Mutant p53 is discussed under pathological conformation because selective recognition of the abnormal conformer is the principal design constraint (13). Amyloid-β (Aβ) is discussed under aggregation because oligomer/fibril state determines recognition and clearance capacity despite its extracellular localization (17, 60). TDP-43 is discussed under spatial mislocalization because preservation of nuclear protein while clearing the cytoplasmic pathological pool defines the therapeutic window (61, 62). EGFR illustrates context-dependent assignment: kinase-domain mutant EGFR is considered under activation/resistance (63), whereas overexpressed or aberrantly presented cell-surface EGFR is considered under spatial-state-directed lysosomal clearance (27).

### 2.1 Elevated protein abundance and aberrant dependency

Elevated protein abundance may arise from gene amplification, increased transcription or translation, impaired degradation, or prolonged protein half-life (1, 3, 35, 64). Other diseases show no marked protein accumulation but remain dependent on the continued presence of a target protein to sustain transcriptional networks, protein complexes, anti-apoptotic states, or metabolic pathways (36, 43, 55). A shared feature of these targets is that the disease phenotype depends on the persistent presence of the intact protein. Reducing protein abundance can therefore suppress both catalytic and non-catalytic functions (42, 54, 55).

#### 2.1.1 Estrogen receptor-α

Estrogen receptor-α (ERα) is a ligand-dependent nuclear receptor and transcription factor that serves as a principal driver of ER-positive breast cancer (65). Prolonged endocrine therapy can select for mutations in the ESR1 ligand-binding domain. Mutations such as Y537S and D538G stabilize the active receptor conformation, allowing transcriptional activity to persist under low-estrogen conditions (66, 67). Changes in ER expression, coregulators, and growth factor signaling can also sustain pathway activity (65). Although ESR1 mutations confer ligand-independent activation, the shared pharmacological dependency of ER-positive breast cancer remains the continued presence of ERα and its transcriptional network (50, 65). We therefore classify ERα within the aberrant-dependency category.

Endocrine therapy for ER-positive breast cancer primarily includes aromatase inhibitors, selective estrogen receptor modulators, and selective estrogen receptor degraders (SERDs) (65). SERDs induce conformational changes in ER through ligand binding and promote receptor downregulation (68). Heterobifunctional ER degraders further recruit endogenous E3 ligases to drive ER ubiquitination and proteasomal clearance (50, 69).

Early studies of fully small-molecule ER PROTACs focused on the target-binding ligand, conjugation site, and linker conformation (69). The VHL-recruiting degrader ERD-308 achieved DC_50_ values of 0.17 and 0.43 nM in MCF-7 and T47D cells, respectively, and reduced ER levels by more than 95% at 5 nM (70). The related compound ERD-148 depleted both unphosphorylated and phosphorylated ERα and retained activity in fulvestrant-resistant cells (71). The subsequent CRBN-recruiting degrader ERD-3111 combined oral exposure with *in vivo* efficacy, reducing ER levels and suppressing tumor growth in models harboring wild-type or mutant ESR1 (72). QDE-003-W, aptamer-based degraders, and hypoxia-responsive molecules further expanded the range of ligand structures, target-recognition mechanisms, and microenvironment-dependent activation strategies (73–75).

Vepdegestrant completed the transition of ER PROTACs from preclinical development to an approved therapy (50). This oral heterobifunctional degrader reduces both wild-type ER and mutant forms such as Y537S and D538G, and has shown activity as monotherapy and in combination regimens in models of ER-positive breast cancer (76). In the phase III VERITAC-2 trial, median progression-free survival was 5.0 months with vepdegestrant and 2.1 months with fulvestrant, among patients with ESR1-mutant disease, with clinical benefit concentrated in this molecular subgroup (50). On May 1, 2026, the FDA approved vepdegestrant for advanced or metastatic ESR1-mutant, ER-positive, HER2-negative breast cancer and simultaneously approved Guardant360 CDx as a companion diagnostic (51).

ERα degradation has thus progressed from efficient cellular clearance and coverage of mutant receptors to an approved oral therapy, with ESR1 mutation status emerging as a key biomarker for patient stratification (50, 51, 76). For transcription-factor dependencies that retain a ligand-accessible domain, clinical translation therefore requires co-optimization of mutant coverage, sustained target depletion, and oral exposure; cellular degradation potency alone is insufficient (56, 57).

#### 2.1.2 Androgen receptor

Androgen receptor (AR) is a central transcription factor in the initiation and progression of prostate cancer (77). Mutations such as H875Y, T878A, and L702H can alter ligand recognition and responses to AR antagonists; some earlier studies referred to these variants as H874Y, T877A, and L701H, respectively (78). Splice variants lacking the ligand-binding domain, including AR-V7, retain nuclear localization under androgen-deprived conditions and continue to drive AR-dependent transcription (79, 80). Although AR mutations and splice variants contribute to persistent activation and treatment resistance, the shared dependency of prostate cancer remains the transcriptional program maintained by AR protein (77, 80). This section therefore focuses on clearance of the full-length receptor and its variants.

Androgen deprivation therapy (ADT), combined with AR pathway inhibitors such as abiraterone and enzalutamide, is a cornerstone of treatment for advanced prostate cancer (81, 82). AR amplification and ligand-binding domain mutations that emerge during disease progression make direct depletion of full-length AR an important strategy for maintaining pathway suppression (77, 78). By contrast, splice variants lacking the ligand-binding domain, including AR-V7, require degraders that recognize retained AR domains (80, 83).

Early AR PROTAC studies examined how the target-binding ligand, E3 ligase ligand, and linker influenced degradation activity (84, 85). The VHL-recruiting degrader ARD-69 achieved DC_50_ values below 1 nM in LNCaP and VCaP cells and reduced AR levels by more than 95% (84). ARD-61 depleted both wild-type AR and mutants such as H875Y and T878A, while retaining activity in models of AR amplification and enzalutamide resistance (86).

Subsequent development of AR degraders shifted from proof of concept toward broader coverage of clinically relevant mutations, improved oral pharmacokinetic properties, and clinical validation. The CRBN-recruiting degrader bavdegalutamide (ARV-110) induces degradation of wild-type AR and ligand-binding domain mutants, including T878A/S and H875Y. Antitumor activity was observed in enzalutamide- or abiraterone-resistant preclinical models and phase I/II studies, although activity against AR L702H and AR-V7 was more limited (87, 88). BMS-986365 combines CRBN-dependent AR degradation with competitive antagonism and is being compared with investigator-selected docetaxel or an alternative AR pathway inhibitor in the randomized phase III rechARge trial (89, 90). HP518 has generated preliminary clinical data from a phase I dose-escalation study but remains in early clinical development (91). AZD9750 was optimized for its AR-binding ligand scaffold and oral exposure and has progressed from preclinical development to the phase I/II ANDROMEDA trial (92, 93).

For splice variants lacking the ligand-binding domain, such as AR-V7, targeting strategies have expanded to the AR amino-terminal domain. The covalent degrader EN1441 binds Cys125 within the amino-terminal intrinsically disordered region, destabilizing full-length AR and AR-V7 and inducing their proteasome-dependent degradation (94). Z15 suppresses signaling driven by wild-type AR, mutant AR, and AR-V7 (95).

Further development of AR degraders should broaden coverage across ligand-binding domain mutations and splice variants such as AR-V7, while incorporating AR amplification, mutation, and splicing status into patient stratification (77, 78, 88). More generally, AR illustrates that target-domain selection is an upstream determinant of resistance coverage: degraders directed only to the ligand-binding domain cannot intrinsically address variants that lack that domain, regardless of subsequent linker or E3-ligase optimization (80, 83, 94, 95).

#### 2.1.3 BET proteins and SMARCA2

The bromodomain and extraterminal (BET) family proteins BRD2, BRD3, and BRD4 regulate chromatin-dependent transcription by recognizing acetylated lysine residues (96). BRD4 also recruits transcription elongation complexes to sustain the expression of proliferation-associated genes such as MYC (97, 98). Some leukemias, lymphomas, and solid tumors develop a persistent dependency on BET-driven aberrant transcriptional programs (96, 97, 99, 100). Conventional BET inhibitors primarily disrupt bromodomain binding to acetylated proteins, whereas targeted degradation removes the intact BET protein together with its non-catalytic regulatory functions (43, 96, 98).

In 2015, dBET1 and ARV-825 established the feasibility of CRBN-recruiting BET degradation in models of leukemia and Burkitt lymphoma, respectively. dBET1 induced degradation of BRD2, BRD3, and BRD4, whereas ARV-825 produced a rapid and sustained reduction in BRD4, advancing the development of fully small-molecule BET PROTACs (43, 101). The VHL-recruiting degrader MZ1, reported in the same year, showed relative selectivity for BRD4 degradation (102). Subsequent structural studies of the ternary complex showed that the folded conformation of MZ1, complex cooperativity, and newly formed protein–protein interactions collectively shaped degradation selectivity (47).

Building on these advances, BET degraders were developed to improve potency, subtype selectivity, and drug-like properties. The CRBN-recruiting degraders BETd-260 and QCA570 achieved potent BET degradation and *in vivo* antitumor activity in leukemia models (103, 104). The trivalent degrader SIM1 engages both bromodomains of BET proteins, increasing binding affinity and ternary-complex stability (105). BD-7148 and BD-9136 use conformational constraints to achieve greater selectivity for BRD4 degradation (106). In addition, the monovalent molecule PLX-3618 promotes the formation of a ternary complex between BRD4 and DDB1- and CUL4-associated factor 11 (DCAF11), inducing BRD4 degradation and supporting the feasibility of direct monovalent BET degradation (107). CR10, which contains a semirigid linker, showed improved pharmacokinetic properties and *in vivo* antitumor activity (108).

SMARCA2 and SMARCA4 are alternative core catalytic ATPase subunits of the SWI/SNF chromatin-remodeling complex (109). Some tumors with SMARCA4 loss depend on SMARCA2 to preserve residual SWI/SNF function, making SMARCA2 a synthetic-lethal target defined by a clear genetic context (110). Because the bromodomains of SMARCA2 and SMARCA4 are highly homologous, selective SMARCA2 degradation can arise from ternary-complex geometry and differences in protein surfaces, rather than from the binding selectivity of the binary ligand (111, 112).

ACBI1 first established the degradability of SMARCA2, SMARCA4, and PBRM1 (113). A947 achieved preferential SMARCA2 degradation despite lacking subtype selectivity in binary binding, whereas ACBI2 combined SMARCA2 selectivity with oral exposure and *in vivo* efficacy (111, 112). SMD-3040 further improved selectivity for SMARCA2 over SMARCA4, while SMD-6346 achieved favorable oral exposure and *in vivo* antitumor activity (114, 115). A phase I study of the VHL-recruiting degrader PRT3789 as monotherapy and in combination with docetaxel has been completed. In patients with SMARCA4-inactivated tumors, treatment reduced SMARCA2 levels in peripheral blood mononuclear cells and showed preliminary antitumor activity (116, 117). In addition, PRT7732, a distinct SMARCA2 degrader developed by the same company, represents a subsequent optimization strategy with oral bioavailability as an important design objective, highlighting the continued refinement of SMARCA2 degraders toward improved drug-like properties (118). A phase II study combining PRT3789 with pembrolizumab was terminated (119).

BET proteins and SMARCA2 represent two distinct degradation requirements: aberrant transcriptional dependency and paralog-based synthetic lethality, respectively (99, 110). BET degradation emphasizes removal of the intact transcriptional regulatory machinery, whereas SMARCA2 degradation relies more heavily on a defined genetic context to create a therapeutic window. For both target classes, degradation potency and selectivity are shaped by ternary-complex geometry. These examples show that degradation selectivity can emerge from the induced ternary complex even when the parent ligand has limited binary selectivity, making protein-surface complementarity and ternary-complex geometry exploitable medicinal-chemistry variables (47, 111, 112).

#### 2.1.4 IKZF1 and IKZF3

IKZF1 and IKZF3 are Ikaros-family zinc-finger transcription factors that regulate lymphocyte differentiation and plasma cell–associated transcriptional programs (40, 120). Multiple myeloma shows a well-established dependency on the aberrant transcriptional network controlled by IKZF1/3, and related dependencies occur in some mature B-cell malignancies (40, 121, 122). The interferon regulatory factor 4 (IRF4)–MYC axis is closely linked to myeloma cell survival (120, 121). Because IKZF1/3 lack defined ligand-binding pockets suitable for conventional occupancy-driven inhibition, induced protein degradation has emerged as a representative pharmacological strategy (40, 41, 123, 124).

Immunomodulatory drugs such as lenalidomide and pomalidomide bind CRBN and alter its substrate-recognition profile, thereby recruiting IKZF1 and IKZF3 for ubiquitination and proteasomal degradation (40, 41, 122). This mechanism explains their suppression of myeloma-associated transcriptional programs and has established the basic paradigm by which molecular glues reprogram E3 ligase substrate selectivity. Subsequent cereblon E3 ligase modulators (CELMoDs), including iberdomide and mezigdomide, further enhanced IKZF1/3 degradation through stronger CRBN binding, substrate recruitment, and degradation efficiency (123, 124). A prespecified interim analysis of the phase III SUCCESSOR-2 trial showed a median progression-free survival of 18.0 months with mezigdomide plus carfilzomib and dexamethasone, compared with 8.3 months in the control group. The risk of disease progression or death was reduced by 52% (HR = 0.48) (125). The interim phase III analysis therefore provides clinical efficacy evidence for CELMoD-mediated IKZF1/3 degradation.

Clinical progress with IKZF1/3 demonstrates that molecular glues can reprogram substrate recognition through compact chemical scaffolds (40, 41, 125). Their long-term efficacy must be evaluated in relation to CRBN expression, E3 ligase complex integrity, and variation at the substrate-binding interface (126–128). For molecular-glue optimization, the relevant structure–activity relationship (SAR) therefore extends beyond ligase affinity to the ligand-shaped protein surface that recruits the neosubstrate, with degradation selectivity and resistance emerging from the complete induced interface (129).

#### 2.1.5 RBM39 and cyclin K

RNA-binding motif protein 39 (RBM39) regulates pre-mRNA splicing and also functions as a transcriptional coactivator (130). Cyclin K forms kinase complexes with cyclin-dependent kinases 12 and 13 (CDK12/13), which regulate transcription elongation and RNA processing and sustain the expression of selected DNA damage response genes (131, 132). Some tumors depend on RBM39-driven splicing programs or cyclin K–CDK12/13-mediated transcription. Their rationale as degradation targets therefore rests primarily on tumor-cell dependency rather than elevated protein abundance (133, 134).

The aryl sulfonamides indisulam, E7820, and tasisulam act as molecular glues that promote ternary-complex formation between RBM39 and the DCAF15-containing CRL4 ligase complex (CRL4^DCAF15^). This interaction induces RBM39 ubiquitination and proteasomal degradation, leading to widespread splicing abnormalities (135). Structural studies showed that degradation depends primarily on interface complementarity between DCAF15 and the second RNA recognition motif of RBM39, rather than on high-affinity binary binding (136). E7820 produced pharmacodynamic evidence of RBM39 depletion and altered splicing in patients with spliceosome-mutant myeloid malignancies, although clinical activity was limited (137). The oral RBM39 degrader ST-01156 has entered a phase I/Ib trial in advanced solid tumors (138).

Cyclin K molecular glues use an interface-remodeling mechanism distinct from classical substrate-receptor recruitment. When CR8 binds CDK12, its solvent-exposed 2-pyridyl group promotes association of the CDK12–cyclin K complex with DDB1. This interaction induces cyclin K ubiquitination and proteasomal degradation, bypassing the requirement for a canonical DCAF substrate receptor within the CRL4 ligase complex (139). Subsequent structural and SAR studies showed that the CDK12 inhibitory activity and cyclin K degradation activity of SR-4835 and related scaffolds can be tuned relatively independently, providing a basis for structure-guided design of this class of molecular glues (140).

RBM39 and cyclin K exemplify two neomorphic interface mechanisms mediated by DCAF15 and DDB1, respectively. Their clinical translation will depend on tumor dependency on the relevant splicing or transcriptional programs, biomarker-based patient stratification, and the tolerance of normal tissues to broad disruption of RNA processing and transcriptional remodeling. Together, these systems show that solvent-exposed substituents, protein-surface complementarity, and induced-interface geometry can be primary SAR variables, and that degradation activity may be optimized partly independently from the occupancy-driven pharmacology of the ligand-bound protein (136, 139, 140).

#### 2.1.6 BCL-XL and MCL1

BCL-XL and MCL1 are anti-apoptotic members of the BCL-2 family. By binding BCL-2 homology 3 (BH3)-only proteins such as BIM and effector proteins such as BAK and BAX, they suppress mitochondrial outer membrane permeabilization and apoptosis (141). Aberrant upregulation of either protein raises the apoptotic threshold of tumor cells and contributes to treatment resistance in leukemia, lymphoma, and diverse solid tumors (142, 143). Development of BCL-XL inhibitors has been limited by mechanism-based thrombocytopenia (144). MCL1 is essential for cardiomyocyte survival and mitochondrial homeostasis, creating cardiac safety risks for systemic inhibition (145, 146).

BCL-XL degraders primarily exploit differences in E3 ligase expression between platelets and target cells to achieve tissue selectivity. The CRBN-recruiting degraders XZ424, XZ739, and PZ15227 and the VHL-recruiting degrader DT2216 were derived from A-1155463 or navitoclax and showed reduced platelet toxicity in their respective models (147–150). XZ424, XZ739, and DT2216 retained or enhanced activity in BCL-XL-dependent tumor models, whereas PZ15227 provided proof of concept mainly in senolytic models. DT2216 exploits the low abundance of VHL in platelets to induce BCL-XL ubiquitination and proteasomal degradation preferentially in tumor cells, producing activity as monotherapy or in combination regimens across multiple xenograft models. The VHL-recruiting degrader 753b targets both BCL-XL and BCL-2, offering a dual-degradation strategy for tumors that depend on both proteins (151).

DT2216 has completed a first-in-human phase I study. At the recommended phase II dose, rapid and sustained BCL-XL degradation was observed in peripheral blood leukocytes. Thrombocytopenia still occurred but was largely confined to the first treatment cycle and recovered rapidly. The clinical pharmacodynamic profile of DT2216 indicates that differential E3-ligase expression can improve tissue selectivity, although it does not fully eliminate on-target platelet toxicity (152). The CRBN-recruiting degrader SIAIS361034 also selectively degrades BCL-XL. In combination with sorafenib, it releases BIM from sequestration by BCL-XL and MCL1, thereby enhancing apoptosis in hepatocellular carcinoma cells and producing synergistic antitumor activity *in vivo* (153).

MCL1 degradation remains largely at the preclinical stage (154, 155). dMCL1-2 uses the MCL1 inhibitor A-1210477 as its target-recognition moiety and recruits CRBN, establishing the feasibility of heterobifunctional small molecules that induce MCL1 degradation through the UPS. C3 was derived from a nonselective MCL1/BCL-2 ligand but selectively degraded MCL1, showing that degradation selectivity can diverge from the binding selectivity of the binary ligand. A 2026 conference abstract reported that CT-03 induced MCL1 degradation in hematologic malignancy models and provided preliminary assessments of combination treatment with venetoclax, intermittent dosing, and cardiac safety (156).

BCL-XL degradation has generated early pharmacodynamic evidence in humans, whereas MCL1 degradation remains at the preclinical stage. Translation of BCL-XL degraders will depend primarily on tissue selectivity and platelet safety (147, 152), while MCL1 degraders require careful evaluation of dosing schedules and cardiac safety (145, 146, 156). Differential E3-ligase abundance can therefore function as a tissue-selectivity filter, but this advantage must be validated against residual on-target toxicity rather than inferred solely from deeper degradation in the desired cell type (147, 152).

#### 2.1.7 Metabolic regulatory proteins: PTP1B, DPP-4, and 11β-HSD1

Protein tyrosine phosphatase 1B (PTP1B), dipeptidyl peptidase-4 (DPP-4), and 11β-hydroxysteroid dehydrogenase type 1 (11β-HSD1) regulate insulin signaling, incretin inactivation, and local glucocorticoid regeneration, respectively. PTP1B negatively regulates insulin signaling, DPP-4 inactivates incretins such as glucagon-like peptide-1 (GLP-1) and glucose-dependent insulinotropic polypeptide (GIP), and 11β-HSD1 regenerates active glucocorticoids within tissues. Increased activity of these proteins is associated with metabolic disorders, including insulin resistance, obesity, and type 2 diabetes (157–159). Development of PTP1B and 11β-HSD1 inhibitors has long focused on enzyme selectivity, tissue exposure, and durability of pharmacodynamic effects, whereas DPP-4 inhibitors are widely used to treat type 2 diabetes. Targeted degradation offers a new strategy to prolong target modulation while affecting both catalytic activity and protein-interaction functions (57).

Compound 75, reported in 2024, is a CRBN-recruiting PTP1B PROTAC containing a relatively long 23-atom polyethylene glycol (PEG) linker. PTP1B degradation increased with treatment duration and depended on PTP1B and CRBN binding, ubiquitination, and the proteasome. In HepG2 cells, compound 75 enhanced insulin receptor substrate 1 (IRS-1)/phosphoinositide 3-kinase (PI3K)/Akt signaling. In an oral glucose tolerance test in mice, the 0–2 h blood glucose area under the curve was reduced to 29% of that in the control group (160).

DeDPP4, reported subsequently, links alogliptin to a CRBN ligand and is a representative DPP-4 degrader. In A549 cells, it achieved more than 80% D_max_. Following a single 10 mg/kg dose in mice, DPP-4 levels declined in the liver and adipose tissue, while increased GLP-1 levels and glucose-lowering effects persisted for approximately 60 h. These findings suggest that targeted degradation may extend the duration of metabolic target modulation (161).

11β-HSD1 degraders were constructed using an AZD8329-derived inhibitor as the target-binding ligand and either CRBN- or VHL-recruiting moieties. The optimized compound H-3-V reduced 11β-HSD1 levels through the UPS and improved glucose tolerance and glucose-stimulated insulin secretion in mice with high-fat diet–induced type 2 diabetes. These findings provide proof of concept for extending pharmacological control of 11β-HSD1 from enzyme inhibition to regulation of protein abundance (162).

Degradation of these metabolic targets remains at the preclinical stage (160–162). Translation of metabolic degraders will require direct demonstration of tissue exposure and target depletion in liver, adipose tissue, and skeletal muscle, together with chronic-dosing pharmacodynamics, compensatory responses, and long-term safety (56, 57). Representative dependency states and the molecular-design principles illustrated by selected degraders are summarized in Figure 3.

**Figure 3:**
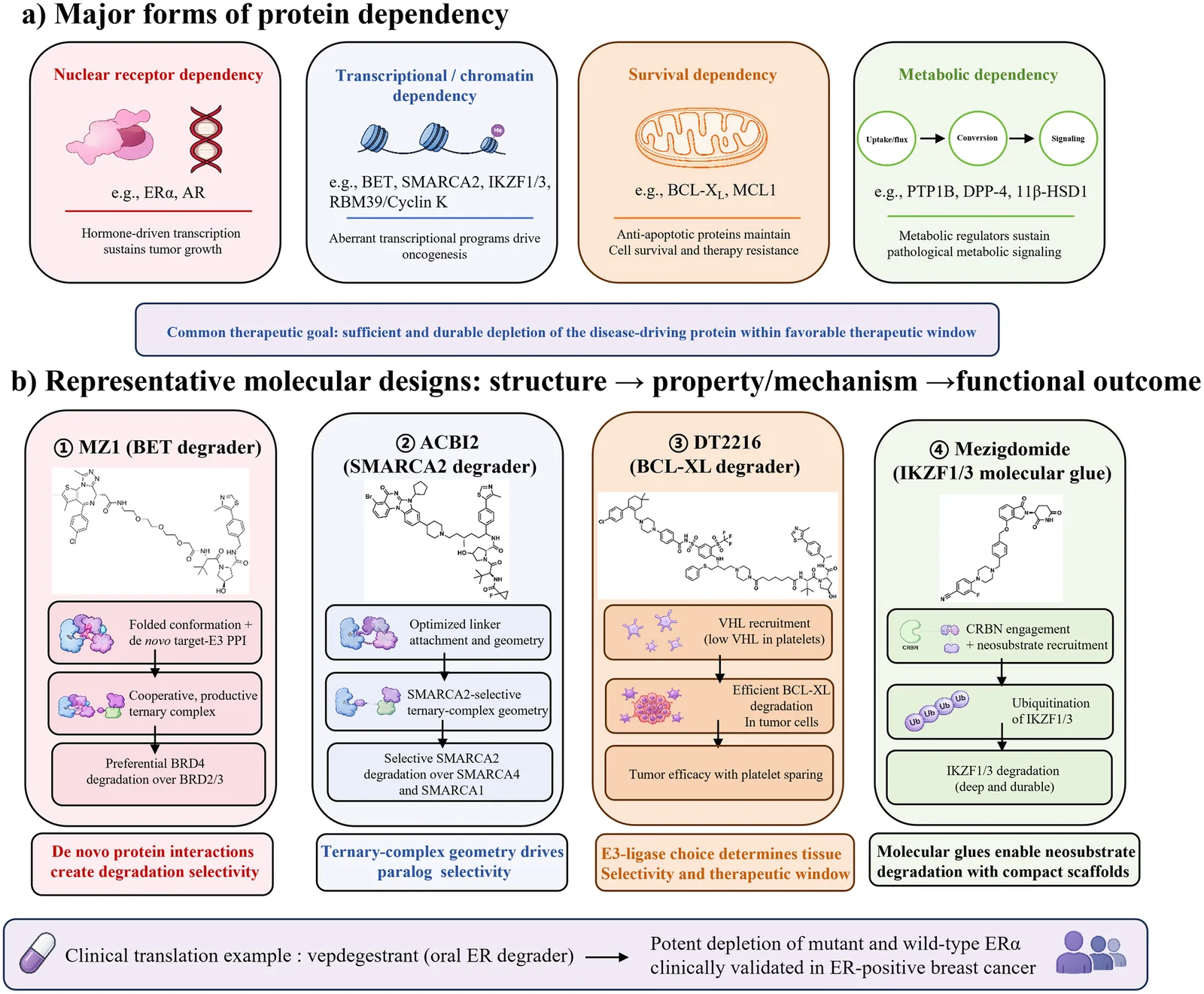
Representative targets and degraders for elevated protein abundance and aberrant dependency. **(a)** Representative disease-driving targets, including ERα, AR, BET proteins, SMARCA2, RBM39/Cyclin K, IKZF1/3, BCL-XL/MCL1, and metabolic regulators, together with their major pathological features and degradation rationale. **(b)** Representative medicinal-chemistry examples—MZ1, ACBI2, DT2216, and mezigdomide—illustrating ternary-complex-derived selectivity, E3-expression-dependent tissue selectivity, and molecular-glue-mediated neosubstrate recruitment.

### 2.2 Emerging degradation modalities beyond classical PROTAC architectures

Although PROTACs represent one of the most extensively developed heterobifunctional degradation platforms, recent advances have expanded the TPD landscape beyond conventional ligand–linker–E3 ligase architectures. These emerging strategies provide alternative solutions for targets that remain challenging for classical PROTAC design, including proteins with limited ligandability, unfavorable physicochemical properties, or disease-associated conformational states that influence molecular recognition. Importantly, these approaches further support the concept that degradation strategy selection should be guided by the biological characteristics and pathological state of disease-associated species rather than by a single technological platform.

#### 2.2.1 Covalent degraders and linker-free PROTACs

Covalent degraders represent an emerging class of targeted degradation molecules that incorporate irreversible target engagement into degradation design (163). Compared with reversible binders traditionally used in PROTACs, covalent warheads can enhance target residence time and stabilize target engagement, potentially increasing the probability of productive degrader complex formation and subsequent target degradation. By stabilizing the target–degrader interaction, covalent degraders may expand the range of proteins accessible to targeted degradation and provide additional opportunities for previously difficult-to-degrade targets (164).

In parallel, linker-free PROTACs have emerged as an alternative strategy to simplify the architecture of heterobifunctional degraders (165). Unlike conventional PROTACs that rely on a chemically optimized linker connecting a target-binding ligand and an E3 ligase recruiter, linker-free designs directly integrate these functional elements into a compact molecular framework. This approach may improve physicochemical properties, simplify molecular architecture, and facilitate optimization of cellular permeability and pharmacological behavior. Together, covalent degraders and linker-free PROTACs represent complementary strategies that address some limitations associated with classical PROTAC architectures while maintaining the principle of induced proximity-driven degradation.

#### 2.2.2 Hydrophobic tagging (HyT)

Hydrophobic tagging (HyT) represents a mechanistically distinct degradation strategy that exploits endogenous protein quality-control pathways rather than relying exclusively on engineered E3 ligase recruitment (166). In HyT approaches, a hydrophobic moiety is attached to a target-binding ligand, introducing an artificial hydrophobic signal that promotes recognition by cellular proteostasis machinery and ultimately facilitates target elimination (167).

Compared with ubiquitin-dependent degradation systems, HyT provides an alternative route for inducing protein clearance and may be particularly relevant for pathological states associated with abnormal protein conformation or impaired proteostasis. Therefore, HyT extends the targeted clearance concept beyond classical ubiquitination-based mechanisms and highlights how intrinsic cellular quality-control systems can be therapeutically redirected.

#### 2.2.3 Small-molecule-induced endogenous degradation

Beyond engineered degraders, recent studies have revealed that some conventional small-molecule inhibitors can induce degradation of their target proteins through endogenous proteolytic mechanisms (168). This finding challenges the traditional distinction between pharmacological inhibition and targeted degradation by demonstrating that ligand binding can simultaneously alter protein stability and functional activity.

For example, certain kinase inhibitors have been shown to promote target protein elimination through native cellular degradation pathways, suggesting that degradation capability may represent an underappreciated property of existing pharmacological agents. These observations broaden the concept of TPD from artificially constructed degrader molecules toward a wider pharmacological framework in which endogenous proteolytic capacity can be harnessed for therapeutic protein elimination.

Collectively, these emerging strategies expand the TPD landscape beyond classical PROTAC and molecular glue approaches. Rather than representing independent technological alternatives, they provide additional mechanisms for matching degradation strategies with specific pathological protein states. Future development of targeted clearance approaches may require integrating target biology, molecular recognition characteristics, and available cellular degradation pathways to identify appropriate therapeutic modalities. Such a pathology-state-guided approach may facilitate rational selection of degradation strategies across diverse disease contexts sharing common biological constraints.

### 2.3 Persistent activation, gain-of-function alterations, and resistance mutations

The pathogenic effects of these proteins arise primarily from activating mutations, stabilization of constitutively active signaling conformations, or resistance mutations selected during therapy, rather than from increased protein abundance. Some mutant proteins also sustain disease signaling through scaffolding functions, protein interactions, or complex assembly. The principal value of targeted degradation lies in removing the intact protein and thereby suppressing both catalytic and non-catalytic functions (12, 36, 55). Degrader design should prioritize clinically relevant variant coverage and, where required for the therapeutic window, selective sparing of the wild-type protein.

#### 2.3.1 KRAS

KRAS is a small GTPase molecular switch that transmits signals from receptor tyrosine kinases to downstream effector pathways, including RAF–MEK–ERK. Hotspot mutations such as G12C, G12D, G12V, and Q61H/L/R impair intrinsic and/or GTPase-activating protein–stimulated GTP hydrolysis, shifting KRAS toward the active GTP-bound state. These mutations drive malignancies including pancreatic cancer, colorectal cancer, and non-small cell lung cancer (NSCLC) (169). The clinical use of KRAS G12C inhibitors established the feasibility of directly targeting mutant KRAS (170). However, resistance can arise through secondary on-target mutations, amplification of the mutant allele, or feedback and bypass reactivation of the receptor tyrosine kinase–RAS–mitogen-activated protein kinase (MAPK) pathway (171, 172).

Early KRAS degradation studies exploited the covalent binding site created by the G12C mutation. The VHL-recruiting degrader LC-2 uses an MRTX849-derived covalent ligand to recognize KRAS G12C. It induced rapid and sustained degradation of the endogenous mutant protein and suppressed MAPK–ERK signaling, establishing the feasibility of clearing membrane-associated endogenous KRAS through the UPS (173). Subsequent biophysical and structural studies of ternary-complex formation, together with linker optimization, expanded allele coverage beyond G12C to multiple oncogenic variants. The VHL-recruiting degrader ACBI3, reported in 2024, efficiently degraded 13 of 17 common oncogenic KRAS alleles. Compared with the corresponding inhibitor, ACBI3 produced deeper and more durable pathway suppression across multiple KRAS-mutant cell models and induced tumor regression in mouse xenograft models (174).

KRAS G12D lacks the cysteine thiol that enables covalent targeting of KRAS ^G12C^. ASP3082 therefore uses a noncovalent KRAS G12D ligand and depends on formation of a productive KRAS G12D–degrader–VHL ternary complex. The VHL-recruiting degrader ASP3082 (setidegrasib) selectively degrades KRAS G12D and induces tumor regression across multiple KRAS G12D-mutant xenograft models (175).

The published phase I study enrolled 203 previously treated patients with KRAS G12D-mutant advanced solid tumors. Setidegrasib was administered intravenously once weekly at doses of 10–800 mg. In the cohort receiving the recommended phase II dose of 600 mg, the objective response rates were 36% in patients with NSCLC and 24% in patients receiving second- or third-line treatment for metastatic pancreatic ductal adenocarcinoma (PDAC) (52). Setidegrasib has entered two phase III trials. One compares it with docetaxel in patients with NSCLC that progressed after platinum-based chemotherapy and immune checkpoint inhibition. The other evaluates setidegrasib combined with modified FOLFIRINOX (mFOLFIRINOX) or NALIRIFOX as first-line treatment for metastatic PDAC.

KRAS degradation has progressed from proof of concept with G12C to late-stage clinical development for G12D and clearance across multiple alleles (52, 173–176). Further development should broaden coverage of secondary mutations, reduce the burden of intravenous administration, and assess how KRAS resynthesis and recovery of bypass signaling affect the durability of pharmacodynamic effects. KRAS therefore illustrates a general separation between recognition chemistry and degradation breadth: the target-binding mode establishes initial allele selectivity, whereas linker and ternary-complex architecture can broaden the spectrum of variants that undergo productive degradation (174, 175).

#### 2.3.2 BTK

Bruton tyrosine kinase (BTK) is a key non-receptor tyrosine kinase in the B-cell receptor signaling pathway and promotes cell survival and proliferation in chronic lymphocytic leukemia, mantle cell lymphoma, and other B-cell malignancies (177). Covalent BTK inhibitors exert their activity through binding to Cys481, and prolonged treatment can select for or enrich resistance mutations such as C481S that impair covalent engagement (178). Noncovalent inhibitor therapy can also select for kinase domain mutations, including V416L, T474I, and L528W, which weaken drug binding while preserving B-cell receptor pathway output (179). Although mutations such as V416L and L528W markedly reduce BTK catalytic activity, downstream signaling can remain active. L528W can additionally activate HCK through non-catalytic protein interactions. Persistence of downstream signaling despite kinase-impairing mutations indicates that intact BTK retains signaling-organizing functions beyond catalytic activity, providing a pharmacological rationale for targeted degradation.

The CRBN-recruiting degrader NX-2127 can degrade wild-type BTK as well as multiple resistant variants, including C481S, V416L, T474I, and L528W. In addition, it induces IKZF1 and IKZF3 degradation through CRBN molecular-glue activity. In a phase I study of chronic lymphocytic leukemia, NX-2127 reduced BTK levels by more than 80%, and early clinical responses were observed in patients harboring different BTK mutations and exhibiting distinct catalytic activity states (180). The NX-2127 clinical pharmacodynamic profile supports elimination of intact BTK as a means of disrupting both catalytic and scaffolding functions.

IKZF1/3 degradation introduces additional immunomodulatory effects, making BTK degradation selectivity an increasingly important consideration in subsequent development. BGB-16673 and bexobrutideg (NX-5948) are both oral BTK-selective degraders. Each has entered phase III randomized studies comparing the degrader with the noncovalent BTK inhibitor pirtobrutinib in patients with relapsed or refractory chronic lymphocytic leukemia/small lymphocytic lymphoma previously treated with covalent BTK inhibitors (181, 182).

BTK degradation may still be subject to target-mediated resistance. A428D can undergo clonal expansion during degrader treatment and confer cross-resistance to multiple classes of covalent BTK inhibitors, noncovalent BTK inhibitors, and clinical-stage BTK degraders. Structural studies indicate that the Asp428 side chain creates steric interference with the ATP adenine ring and adenine-mimetic groups present in multiple BTK-targeting molecules, thereby reducing target engagement and preventing subsequent degradation. Therefore, clinical application will require integration of prior BTK-targeted treatment history with dynamic mutation monitoring, together with continued development of recognition scaffolds that employ alternative binding modes or avoid the A428-adjacent region. Accordingly, degrader robustness requires diversity in target-recognition geometry as well as in the recruited E3 ligase; reuse of closely related inhibitor-derived binding motifs can transmit the same target-site resistance liability across both occupancy-driven inhibitors and degraders (183).

#### 2.3.3 Proteasomal degradation of mutant EGFR

Epidermal growth factor receptor (EGFR) is a transmembrane receptor tyrosine kinase. Activating mutations such as exon 19 deletions and L858R stabilize the active conformation of the receptor kinase and continuously drive downstream signaling (63). T790M enhances ATP competition and confers resistance to first- and second-generation EGFR tyrosine kinase inhibitors; C797S disrupts the covalent interaction between third-generation inhibitors and Cys797 and represents a major on-target mechanism of acquired osimertinib resistance (184, 185). Reported small-molecule EGFR degraders generally conjugate ligands that recognize intracellular ATP-binding sites or allosteric pockets with E3 ligase ligands, thereby inducing full-length EGFR degradation and suppressing persistent downstream signaling output (186, 187).

The allosteric degrader DDC-01-163 selectively reduces L858R/T790M EGFR and covers some resistant variants containing C797S or L718Q, while having limited effects on wild-type EGFR. Subsequent studies expanded the mutation spectrum to include L858R/T790M/C797S and exon 19 deletion–associated variants. The CRBN-recruiting degrader HBE-843, reported in 2026, reduced multiple activating or resistant EGFR variants while maintaining selectivity over wild-type receptor in corresponding models (188). The EGFR series further shows that similar target-binding ligands can yield distinct mutant-degradation profiles when linker attachment and ternary-complex geometry differ.

CFT8919 is an oral allosteric degrader designed against EGFR L858R, and preclinical studies suggest that it may cover certain T790M- or C797S-containing backgrounds. The compound remains in a phase I study in Greater China, with no complete human pharmacodynamic or efficacy data currently available. The developer has decided not to advance further clinical development outside Greater China at this time (189–191).

The major challenges for EGFR degradation are expanding coverage of compound mutations while preserving wild-type receptor selectivity. Drug exposure in brain metastases, receptor resynthesis, and bypass signaling activation such as MET pathway reactivation will collectively determine its therapeutic applicability in EGFR-mutant NSCLC (35, 57). EGFR thus highlights a recurring medicinal-chemistry trade-off in mutant-selective degradation: broader mutation coverage, wild-type sparing, and tissue exposure must be optimized together because improvements in one dimension may narrow the therapeutic window in another.

#### 2.3.4 IRAK4, RIPK1, and RIPK2

IRAK4, RIPK1, and RIPK2 connect IL-1 receptor/Toll-like receptor (IL-1R/TLR), tumor necrosis factor receptor (TNFR), and NOD receptor signaling pathways, respectively. Their pathogenic roles involve both kinase activity and functions in organizing signaling complexes. Persistent innate immune signaling contributes to inflammatory diseases and tumor immune regulation; therefore, the therapeutic value of degrading these targets mainly lies in simultaneously eliminating both catalytic and scaffolding functions (192–194). Although these proteins may also exhibit misfolding, mutations, post-translational modifications, or abnormal localization, they are grouped here because persistent pathway activation and dependence on the intact kinase/scaffolding protein, rather than protein abundance or aggregation state, principally determine the rationale for degradation and the relevant pharmacodynamic readouts.

The CRBN-recruiting degrader KT-474 (SAR444656) is an oral IRAK4 degrader. In a phase I study, repeated dosing reduced peripheral blood IRAK4 levels by more than 95% on average in healthy participants and patients with atopic dermatitis or hidradenitis suppurativa, with target reduction and inflammatory pathway changes also observed in lesional skin. Subsequent phase II development was not continued. The KT-474 study demonstrates that deep IRAK4 depletion is achievable in humans, although target reduction alone did not establish therapeutic benefit (193, 195).

RIPK2 degraders have employed different E3 ligase recruitment strategies, including VHL, IAP, and CRBN recruitment (196). Some optimized compounds achieve sustained degradation in peripheral tissues of animal models, with pharmacodynamic effects lasting longer than measurable drug exposure. In an ischemic stroke model reported in 2026, peripheral RIPK2 reduction was associated with inflammatory modulation and partial improvement in neurological function; however, limited degradation within the brain suggested that tissue accessibility may restrict the selection of therapeutic indications (197).

RIPK1 degradation studies have primarily focused on its non-catalytic scaffolding function. The VHL-recruiting degrader LD4172 reduces RIPK1 levels, promotes immunogenic cell death, and enhances tumor responses to immunotherapy (192). The optimized compound LD5097 achieved greater metabolic stability, *in vivo* exposure, and RIPK1 degradation in tumor tissue through modifications to the linker, target-ligand attachment orientation, and VHL ligand (198).

Translational requirements for these inflammatory signaling proteins vary by indication. Chronic inflammatory diseases require careful control of the depth and tissue distribution of long-term immune modulation (193, 196, 197). In cancer, development must distinguish patterns of target dependency across tumor cells, immune cells, and normal tissues (192, 198). More generally, when disease signaling depends on both catalytic and scaffolding functions, degradation can offer a mechanistic advantage over kinase inhibition, but the medicinal-chemistry objective becomes controlled removal of the complete signaling node in the disease-relevant tissue rather than indiscriminate maximal depletion (192–194).

#### 2.3.5 LRRK2

Leucine-rich repeat kinase 2 (LRRK2) is a multidomain protein with kinase, GTPase, and scaffolding functions (199). G2019S increases kinase activity, whereas variants such as R1441C/G/H disrupt GTPase regulation and Rab protein phosphorylation; these mutations are closely associated with inherited Parkinson's disease (200). Compared with kinase inhibition alone, degradation can simultaneously eliminate the multidomain interactions of LRRK2. This approach, however, must overcome the limited cell permeability and blood–brain barrier (BBB) exposure of heterobifunctional molecules (201).

The VHL-recruiting degrader XL01126 uses optimized linker connectivity and ternary-complex cooperativity to induce rapid LRRK2 degradation and suppress Rab10 phosphorylation in cells. In mice, the compound showed oral bioavailability and brain exposure, providing a preclinical example of a brain-penetrant PROTAC (201).

The oral degrader ARV-102 has advanced into human studies. Phase I data disclosed by the developer in 2026 showed that, after 28 days of dosing in patients with Parkinson's disease, cerebrospinal fluid LRRK2 levels declined by approximately 50% or more across dose groups by day 14 and remained reduced through day 28. Changes were also observed in selected endolysosomal and neuroinflammatory biomarkers. These findings provide human pharmacodynamic evidence of target depletion in the central nervous system (CNS). Because the available data derive mainly from company and conference disclosures, they are not yet sufficient to determine whether ARV-102 improves clinical symptoms or slows disease progression (202).

Evaluation of LRRK2 degradation has shifted from cellular potency to brain exposure, target depletion in humans, and long-term safety. Further clinical translation requires definition of responsive populations across LRRK2 variants and sporadic Parkinson's disease, together with continued assessment of pulmonary, renal, immune, and lysosomal consequences of sustained protein reduction (199, 203, 204). For CNS degraders, once cellular degradation is established, linker and whole-molecule optimization must preserve productive degradation while achieving sufficient brain exposure, so central target depletion becomes more informative than cellular DC_50_ alone (201, 202).

Across these examples, degrader performance is determined not only by recognition of the disease-driving variant but also by ternary-complex architecture and whole-molecule properties. Covalent or noncovalent target recognition can define mutation selectivity, whereas linker and ternary-complex optimization can broaden allele or resistance coverage, and successful translation additionally requires adequate exposure in the disease-relevant tissue (Figure 4) (174, 175, 187, 201).

**Figure 4:**
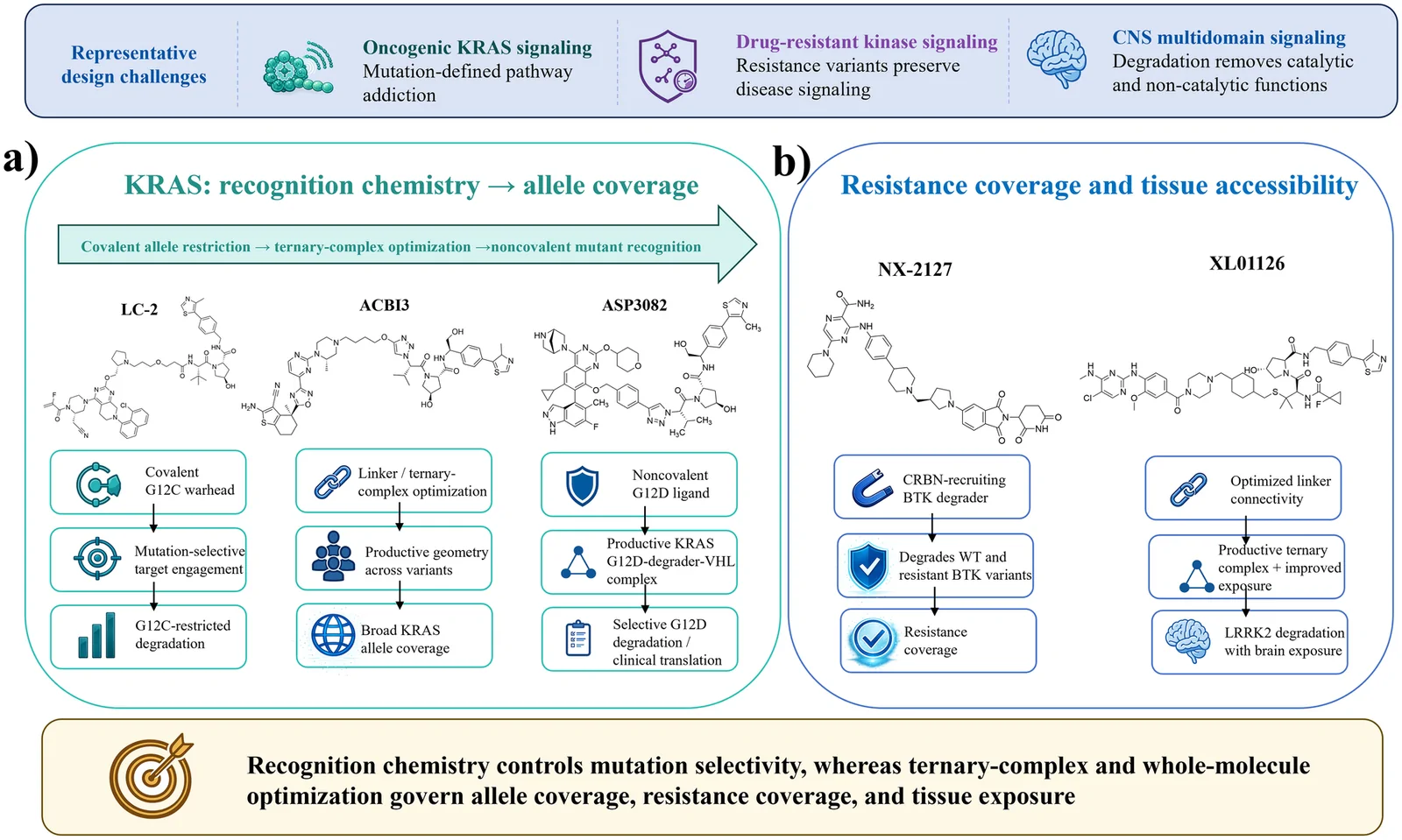
Molecular design principles for degrading persistently activated and drug-resistant proteins. **(a)** Representative KRAS degraders illustrate how recognition chemistry and ternary-complex optimization shape mutant selectivity and allele coverage: LC-2 uses covalent G12C recognition, ACBI3 broadens oncogenic KRAS coverage through linker/ternary-complex optimization, and ASP3082 achieves selective G12D degradation through noncovalent recognition and productive VHL ternary-complex formation. **(b)** NX-2127 illustrates degradation across inhibitor-resistant BTK variants, whereas XL01126 demonstrates how linker and whole-molecule optimization can combine productive LRRK2 degradation with brain exposure. Together, these examples show that recognition chemistry controls mutation selectivity, while ternary-complex and whole-molecule optimization govern allele coverage, resistance coverage, and tissue accessibility.

### 2.4 Protein misfolding and pathological conformations

Point mutations, post-translational modifications, or abnormal metal-ion binding can disrupt native protein folding, expose new hydrophobic surfaces or conformational epitopes, and alter protein half-life and interaction networks (2, 16, 17). For these targets, the design objective is to recognize and eliminate pathological conformations while preserving normal protein function. The solubility and degree of aggregation of the target species further influence selection of the clearance pathway (17, 23).

#### 2.4.1 Mutant p53

Although mutant p53 may simultaneously exhibit gain-of-function activity and abnormal accumulation, it is classified here because the principal design bottleneck for the degradation strategies discussed is selective recognition of the pathological conformation while sparing wild-type p53 (13, 205).

TP53 missense mutations include conformational mutations that destabilize the core domain and contact mutations that impair DNA binding (205). Variants such as R175H and Y220C cause local or global folding defects, whereas R248Q and R273H primarily disrupt DNA binding. Many mutant proteins accumulate in tumor cells because of impaired turnover and promote tumor progression through dominant-negative or gain-of-function effects (13, 205). Current strategies include restoring mutant conformations, modulating chaperone dependence, and reducing TP53 expression (13, 205). However, differences in the protein surfaces created by individual mutations continue to limit the development of broadly selective interventions (205).

Mutant p53 degradation first requires conformation-selective recognition elements. The RNA aptamer-based PROTAC dp53m-RA links an R175H-specific aptamer to a CRBN ligand, inducing ubiquitination and proteasomal degradation of p53-R175H while sparing wild-type p53 and other hotspot mutants (206). The DNA-based dp53 m, generated through aptamer affinity optimization, reduced mutant protein levels in R175H-driven cell and xenograft models and enhanced the response to cisplatin (207). Peptide-based PROTACs constructed from mutant-binding peptides and VHL ligands further show that the same pathological conformation can be directed to the proteasome through distinct recognition elements (208).

Abnormal conformations can also be cleared through the autophagy–lysosomal pathway. A biomimetic nanoreceptor carrying both a mutant p53-binding peptide and a cationic lipid that promotes autophagosome formation directed mutant p53 to autophagy–lysosomal clearance and suppressed tumor growth in ovarian cancer xenograft and patient-derived models (209). A controllable degradation model reported in 2025 showed that reducing the ratio of p53-R248Q to wild-type p53 restored the transcriptional activity of the latter, providing a mechanistic rationale for tumors that retain a wild-type allele. However, this study used an exogenous degradation tag and did not generate a directly translatable drug molecule (210).

Mutant p53 degradation has now been explored using aptamer-based PROTACs, peptide-based PROTACs, and autophagy-directed nanoreceptors, but all remain at the preclinical stage (206–209). A central challenge is to develop deliverable, drug-like ligands that recognize mutant conformations and eliminate gain-of-function proteins while sparing wild-type p53 (205–209). Conformational heterogeneity among hotspot mutations also requires patient stratification at the level of the specific allele (205). Pathological-conformer recognition is therefore an upstream design requirement: targeted degradation cannot compensate for a recognition element that lacks sufficient mutant or conformational selectivity without risking depletion of physiologically important wild-type protein (205–209).

#### 2.4.2 SOD1

Mutations in copper–zinc superoxide dismutase 1 (SOD1) can impair metal binding, dimer stability, or overall folding, promoting the formation of soluble misfolded species and subsequent aggregates (211). This process is a major pathogenic basis of a subset of familial amyotrophic lateral sclerosis (212). Existing protein-lowering strategies mainly use antisense oligonucleotides to reduce SOD1 synthesis, whereas TPD seeks to recognize misfolded conformations at the protein level (213). Because normal SOD1 performs an essential antioxidant function, an ideal degrader should avoid indiscriminate depletion of total SOD1 and achieve effective delivery to both motor neurons and glial cells (211, 214).

MisfoldUbL, reported in 2025, is a BioPROTAC designed to target misfolded SOD1 (215). It consists of an E3 ligase functional domain fused to a single-chain antibody fragment that recognizes the pathological conformation (215). By combinatorial screening of intracellular antibody fragments and E3 ligase modules, the study identified candidates that reduced multiple disease-associated SOD1 variants and intracellular aggregates while largely sparing normal SOD1 (215). Mechanistic experiments showed that both proteasomal and lysosomal pathways contributed to clearance (215). Transgenic expression of MisfoldUbL in SOD1-G93A mice reduced insoluble SOD1 accumulation in the brain, preserved motor neurons and neuromuscular junctions, and delayed disease progression, providing *in vivo* proof of concept for a conformation-selective BioPROTAC (215).

This study also defines the translational boundaries of SOD1 degradation. MisfoldUbL currently depends on gene expression and is not a directly administered small-molecule or protein therapeutic (215). Delivery to the CNS, coverage across SOD1 variants, and the immunological and safety risks of long-term expression remain unresolved (215). SOD1 illustrates that category assignment can shift with pathological progression: soluble misfolded species are primarily a conformational-recognition problem, whereas higher-order oligomers and insoluble aggregates increasingly become a substrate-scale and clearance-capacity problem that may favor autophagy–lysosomal clearance (23, 215). SOD1 therefore illustrates that conformer selectivity and therapeutic delivery are co-limiting variables; successful intracellular recognition by a genetically encoded degrader establishes mechanistic feasibility but does not by itself establish the drug-like tractability of the corresponding clearance strategy (215).

Studies of p53 and SOD1 reveal four major bottlenecks in the degradation of misfolded proteins: the druggability of ligands that recognize pathological conformations, coverage across distinct mutations or conformational subtypes, delivery to disease-relevant tissues, and preservation of normal protein function (206–211, 215). Current evidence remains largely preclinical (206–209, 215). Together, these factors will determine whether conformation-selective degradation can achieve an acceptable therapeutic window.

### 2.5 Protein oligomerization, fibril formation, and insoluble aggregation

Proteins associated with neurodegenerative diseases often progress from misfolded monomers to soluble oligomers, protofibrils, mature fibrils, and inclusion bodies (18, 216). These pathological species differ in toxicity, spatial localization, and susceptibility to cellular clearance (18, 23, 216). This section therefore distinguishes soluble proteins, low-order oligomers, and insoluble aggregates and compares the suitability of different clearance modalities for each pathological species.

#### 2.5.1 Aβ protein

Aβ is classified primarily by aggregation state because species scale determines recognition and pharmacodynamic assessment, whereas extracellular localization acts as a secondary spatial constraint that restricts the available clearance machinery to receptor-mediated pathways (23, 27, 216). Differences in aggregation scale directly affect the choice of recognition element, clearance pathway, and pharmacodynamic assessment (216). The extracellular localization of Aβ further restricts the available clearance machinery, making receptor-mediated endocytic–lysosomal pathways more suitable than cytosolic proteasomal clearance (23, 27).

Aβ is generated by sequential cleavage of amyloid precursor protein (APP) by β- and γ-secretases and is found mainly in the extracellular space and the endocytic–endosomal system, whereas pathological tau accumulates primarily within neurons (20, 216). Mature extracellular Aβ has limited access to cytosolic E3 ligases and the proteasome (23, 27). Reducing APP or BACE1 mainly decreases Aβ production but does not directly eliminate pre-existing oligomers and deposits (216). Classical PROTACs are therefore not the preferred clearance modality for extracellular Aβ (27).

KPLYs, exemplified by KPLY2, reported in 2023, integrates recognition of Aβ aggregates, nanodelivery, and receptor-mediated lysosomal clearance within a single platform (217). It uses a Pittsburgh compound B–derived moiety to recognize Aβ fibrils and a polydopamine nanocarrier to improve brain delivery (217). In the copper-rich microenvironment associated with Aβ deposits, the precursor components assemble *in situ* into an active click-LYTAC, which recruits CD206 and promotes endocytic–lysosomal clearance of Aβ oligomers and fibrils (217). KPLY provides proof of concept for lesion-activated LYTACs targeting extracellular amyloid aggregates. However, its activity depends on multicomponent assembly, nanocarrier distribution, and the local chemical environment. Its pharmacokinetic behavior, manufacturing consistency, and long-term immune effects therefore differ from those of structurally defined small-molecule degraders (217).

SPYTAC, reported in 2026, is a fully synthetic bispecific peptide that binds Aβ at one end and low-density lipoprotein receptor-related protein 1 (LRP1) at the other (218). In this system, LRP1 mediates transcytosis across the BBB, cellular uptake, and lysosomal trafficking, enabling SPYTAC to promote Aβ clearance in both peripheral and central compartments (218, 219). In 5×FAD mice, SPYTAC reduced peripheral and cerebral Aβ burden and was associated with attenuated synaptic damage and improved cognitive performance, providing *in vivo* proof of concept for fully synthetic peptide-mediated degradation of extracellular proteins (218).

Translation of these extracellular Aβ clearance platforms depends on the expression, internalization, and recycling efficiency of transport receptors such as LRP1 and CD206 at the BBB and in neurons and glial cells (217–219). Receptor saturation and competition from endogenous tissue substrates may further limit their activity (217–219). Recognition elements also differ in affinity for soluble monomers, toxic oligomers, protofibrils, and mature deposits (216–218). Each pathological species should therefore be quantified separately, rather than using total Aβ reduction as a surrogate for species-selective clearance (216–218). For extracellular aggregates, receptor trafficking capacity and pathological-species recognition may therefore dominate over classical cellular degrader potency, and total Aβ reduction should not be used as a substitute for demonstrating selective removal of the disease-relevant aggregate species (216–219).

### 2.5.2 Tau

Tau normally contributes to axonal microtubule stability and cytoskeletal regulation (220). Abnormal phosphorylation, truncation, and conformational changes can promote its assembly into soluble oligomers, paired helical filaments, and neurofibrillary tangles (221–223). Tau aggregation occurs across multiple tauopathies, including Alzheimer's disease, frontotemporal lobar degeneration, and progressive supranuclear palsy (224, 225). Tau isoforms, post-translational modifications, and regional brain distribution differ markedly among these disorders (224–226).

TH006 is an early peptide-based degradation chimera developed for tau clearance. It comprises a tau-recognition sequence, a VHL-recruiting sequence, and a cell-penetrating peptide, and promotes tau polyubiquitination and proteasomal degradation (227). TH006 reduced tau levels in cultured neurons and mouse models of Alzheimer's disease and attenuated Aβ-associated cytotoxicity, providing early proof of concept for TPD in the nervous system (227). Its peptide-based structure, however, poses challenges for metabolic stability, cellular uptake, brain distribution, and long-term administration, restricting direct translation into a conventional drug modality.

QC-01-175 links a T807-derived tau positron emission tomography (PET) ligand scaffold to a CRBN ligand (228). It induces proteasome-dependent reduction of total tau and selected phosphorylated tau species in patient-derived neurons carrying A152T or P301L and shows greater degradation activity in pathological neurons than in healthy control neurons (228). These findings suggest that even when the target-binding ligand lacks strict mutation selectivity, the conformation and modification state of pathological tau, together with the cellular proteostasis environment, may confer preferential degradation (228).

The VHL-recruiting degrader C004019 reduced tau levels in cells, hTau mice, and 3×Tg-AD mice and was associated with improvements in selected measures of synaptic function and cognition (229). Its effects were generally greater for soluble tau than for insoluble tau fractions. The investigators therefore proposed that the reduction in insoluble tau may partly reflect a shift in the aggregation–disaggregation equilibrium after depletion of the soluble tau pool, rather than direct proteasomal processing of intact fibrils (229). A decline in the insoluble fraction alone is therefore insufficient to establish direct proteasomal degradation of mature fibrils. Such conclusions require complementary analyses of aggregate turnover, seeding activity, and pathway inhibition.

For higher-order tau aggregates, the mechanism of autophagy-targeting chimeras (AUTOTACs) is better matched to their physical state (230). PBA-1105, PBA-1106, and Anle138b-F105 bind abnormal tau through aggregate-recognition moieties and recruit the ZZ domain of p62/SQSTM1, promoting p62 oligomerization, cargo sequestration, and autophagy–lysosomal clearance (230). Anle138b-F105 reduced tau P301L, oligomeric tau, and phosphorylated tau at nanomolar concentrations in cellular models, supporting the use of selective autophagy to eliminate pathological species that are poorly processed by the proteasome (230).

T3, reported in 2024, uses a proteasome-directed dual-target PROTAC design to reduce both α-synuclein aggregates and total tau, with corresponding DC_50_ values of approximately 1.57 and 4.09 μmol/L (231). The compound produced neuroprotective effects and improved motor phenotypes in MPTP-treated mice (231). However, its micromolar potency, dual-target pharmacology, and evaluation in a non-tauopathy model make it difficult to determine the independent contribution of tau clearance to the overall pharmacodynamic effects (231).

Overall, proteasome-directed PROTACs are better suited to soluble tau pools or tau species that can be unfolded and processed, whereas AUTOTACs are more compatible with oligomers and insoluble aggregates (227–231). Receptor-mediated lysosomal recruitment could, in principle, clear extracellular tau seeds released during intercellular propagation, but cannot replace clearance of the major intracellular pathological tau pool in neurons (27, 49, 232). In published primary studies, evidence for direct tau clearance has come mainly from heterobifunctional PROTACs and AUTOTACs. Direct tau-targeting molecular glues with comparable mechanistic and *in vivo* evidence have yet to be reported (227–231). Tau consequently illustrates that clearance-route selection should follow substrate scale: the same protein can require different molecular architectures for soluble, oligomeric, and insoluble states, making pathological-species definition an upstream medicinal-chemistry decision.

#### 2.5.3 α-Synuclein

α-Synuclein is present mainly as an intrinsically disordered monomer at presynaptic terminals (233). SNCA amplification, increased expression, and certain point mutations promote its assembly into oligomers, protofibrils, and Lewy-related aggregates (234, 235). This pathology occurs in Parkinson's disease, dementia with Lewy bodies, and multiple system atrophy (236). Because normal α-synuclein contributes to the regulation of synaptic vesicles and membrane dynamics, an ideal degrader should preferentially eliminate aggregated species while avoiding sustained depletion of the entire monomer pool (237).

ATC161 is an AUTOTAC designed to target α-synuclein aggregates and comprises an Anle138b-derived aggregate-recognition module linked to a ligand for the p62 ZZ domain (238). It reduced insoluble α-synuclein in a p62- and autophagy–lysosome-dependent manner, with a DC_50_ of approximately 100 nmol/L, while exerting little effect on soluble monomers (238). In mice injected with preformed fibrils, oral ATC161 reduced α-synuclein aggregation and spread across brain regions, attenuated glial responses, and improved motor phenotypes, providing *in vivo* evidence for aggregation-state-selective autophagic degradation (238).

Autophagy-dependent protein reduction alone is insufficient to establish that a degrader acts by directly bridging the target protein to LC3/GABARAP (239). Direct binding of some early ATTEC ligands to LC3/GABARAP has not been consistently reproduced in independent studies (239). ATTEC-based strategies should therefore validate the degradation phenotype, autophagy–lysosomal dependence, and molecular-bridging mechanism separately (239). In addition to assays of autophagic flux and pathway inhibition, orthogonal methods—including direct binding assays, target-occupancy measurements, structural analysis, and key-site mutagenesis—are required to confirm formation of the target protein–degrader–LC3/GABARAP complex (239).

Aptamer-ATTEC links an α-synuclein aptamer to a coumarin-derived small molecule proposed to bind LC3 and induces autophagy-dependent reduction of α-synuclein in cells (240). The aptamer improves the programmability of target recognition, but its cellular uptake, endosomal escape, nuclease stability, and brain delivery differ from those of conventional small molecules. Current evidence also remains largely limited to cellular models (240). Direct recruitment of LC3 requires further validation according to the criteria outlined above (239).

Arg-PEG1-Tα-syn, reported in 2025, uses a benzothiazole-derived moiety to recognize α-synuclein and an arginine group to recruit UBR1, an E3 ligase associated with the N-degron pathway (241). The molecule reduced wild-type and A53T α-synuclein through the UPS and decreased aggregates and associated toxicity in cellular and nematode models (241). This strategy broadens the range of E3 ligases available for TPD but does not confer strict selectivity for aggregated species (241). Data on mammalian brain exposure, target-protein pharmacodynamics, and long-term preservation of normal monomers are also lacking (241).

Dual-target PROTACs exemplified by T3 can also reduce α-synuclein aggregates and tau through the UPS and confer neuroprotection in MPTP models (231). MPTP models primarily capture acute dopaminergic neurotoxicity, whereas preformed fibril injection models emphasize propagation initiated by exogenous seeds (242, 243). Neither fully recapitulates the chronic proteostasis disruption that develops in sporadic Parkinson's disease (242, 243). Proteasome-directed degradation may therefore be prioritized for soluble monomers and low-order oligomers, whereas mature fibrils and larger aggregates are better suited to selective autophagy modalities such as AUTOTACs (231, 238, 241). The final choice should integrate selectivity for pathological species, brain exposure, and preservation of the normal protein pool (231, 237, 238, 241). For aggregation-prone CNS targets, design must balance pathological-species preference, brain exposure, and clearance-pathway capacity. Maximizing bulk protein depletion without distinguishing the normal soluble pool may narrow rather than widen the therapeutic window (237, 238).

#### 2.5.4 Mutant huntingtin and other polyglutamine proteins

Huntington's disease is caused by CAG repeat expansion in the HTT gene (244). The expanded polyglutamine tract alters the conformation of mutant huntingtin (mHTT), promoting the formation of soluble oligomers, fibrils, and nuclear or cytoplasmic inclusions (245, 246). Wild-type HTT contributes to neurodevelopment, vesicular and axonal transport, and cellular homeostasis (247–249). Reducing total HTT may therefore compromise its normal functions, making allele or pathological-state selectivity a central requirement for this target (48, 247–249).

HTT–LC3-linking compounds such as 10O5, 8F20, AN1, and AN2 have been proposed as mHTT-selective ATTECs. These molecules were reported to recognize both expanded polyglutamine (polyQ) tracts and LC3, preferentially reduce mHTT, decrease aggregation, and improve selected disease phenotypes in patient-derived cells, *Drosophila,* and mouse models (48). Some compounds also reduced mutant ATXN3, suggesting that recognition of expanded polyQ conformations may extend to other polyglutamine disorders, including spinocerebellar ataxias (48). However, direct LC3 binding and ternary-complex formation remain subject to the validation concerns outlined above (239). Autophagy-dependent reduction of mHTT alone is insufficient to establish a direct ATTEC-bridging mechanism (239).

AUTOTACs recruit autophagy through a mechanism distinct from direct LC3 bridging (230). PBA-based molecules and Anle138b-F105 recognize abnormal or aggregated conformations such as HttQ97 and reduce mHTT aggregates through p62-mediated selective autophagy, while largely sparing short-polyQ proteins such as HttQ25 (230). This modality derives pathological-state selectivity mainly from neo-surfaces exposed by abnormal aggregates, rather than relying solely on the sequence of the mutant allele (230).

PROTAC 2′, reported in 2026, comprises an amyloid aggregate-recognition ligand, a PEG linker, and the CRBN ligand pomalidomide (250). It reduced mHTT aggregates in cells while largely preserving wild-type HTT (250). In R6/2 mice, continuous delivery by osmotic pump reduced cerebral mHTT aggregation and neuroinflammation and was associated with improvements in body weight, motor coordination, and survival (250). PROTAC 2′ therefore provides proof of concept that, under appropriate ligand- and context-dependent conditions, a UPS-directed degrader can reduce the mHTT aggregate burden *in vivo* (250).

However, the observed reduction in aggregate burden does not by itself demonstrate direct proteasomal processing of mature inclusions. The effect may instead arise, at least in part, from clearance of soluble precursors, shifts in the aggregation–disaggregation equilibrium, or secondary activation of cellular quality-control pathways (230, 250). Moreover, the R6/2 model expresses a truncated HTT exon 1 fragment and shows rapid disease progression (251). Continuous delivery by osmotic pump also differs from long-term oral or intermittent dosing in clinical practice (250). The results therefore cannot be directly extrapolated to the chronic pathology driven by full-length mHTT in adult patients (250, 251).

The primary design variable for TPD of aggregation-prone proteins is the target pathological species, rather than the identity of the protein itself. Although Aβ, tau, α-synuclein, and mHTT involve distinct proteins and diseases, their clearance strategies must distinguish among soluble monomers, toxic oligomers, fibrils, and inclusions (48, 217, 227–231, 238, 240, 241, 250). Clearance efficiency, preservation of normal protein, and functional benefit should be compared within the same pathological species and disease-relevant models (48, 230, 238, 250). The conformation, scale, and spatial localization of pathological species can thus provide a common framework for selecting clearance modalities across proteins (48, 230, 231, 238, 250). Brain exposure, clearance-system capacity, and the translational relevance of disease models remain shared bottlenecks (231, 238, 241, 250, 251). A further mechanistic lesson is that aggregate reduction alone does not establish direct processing of mature aggregates; medicinal-chemistry evaluation should pair species-resolved measurements with pathway-blockade experiments to distinguish clearance of aggregates from depletion of soluble precursors or shifts in aggregation equilibrium (230, 239, 250). These relationships between pathological species, substrate scale, and clearance-route selection are summarized in Figure 5.

**Figure 5:**
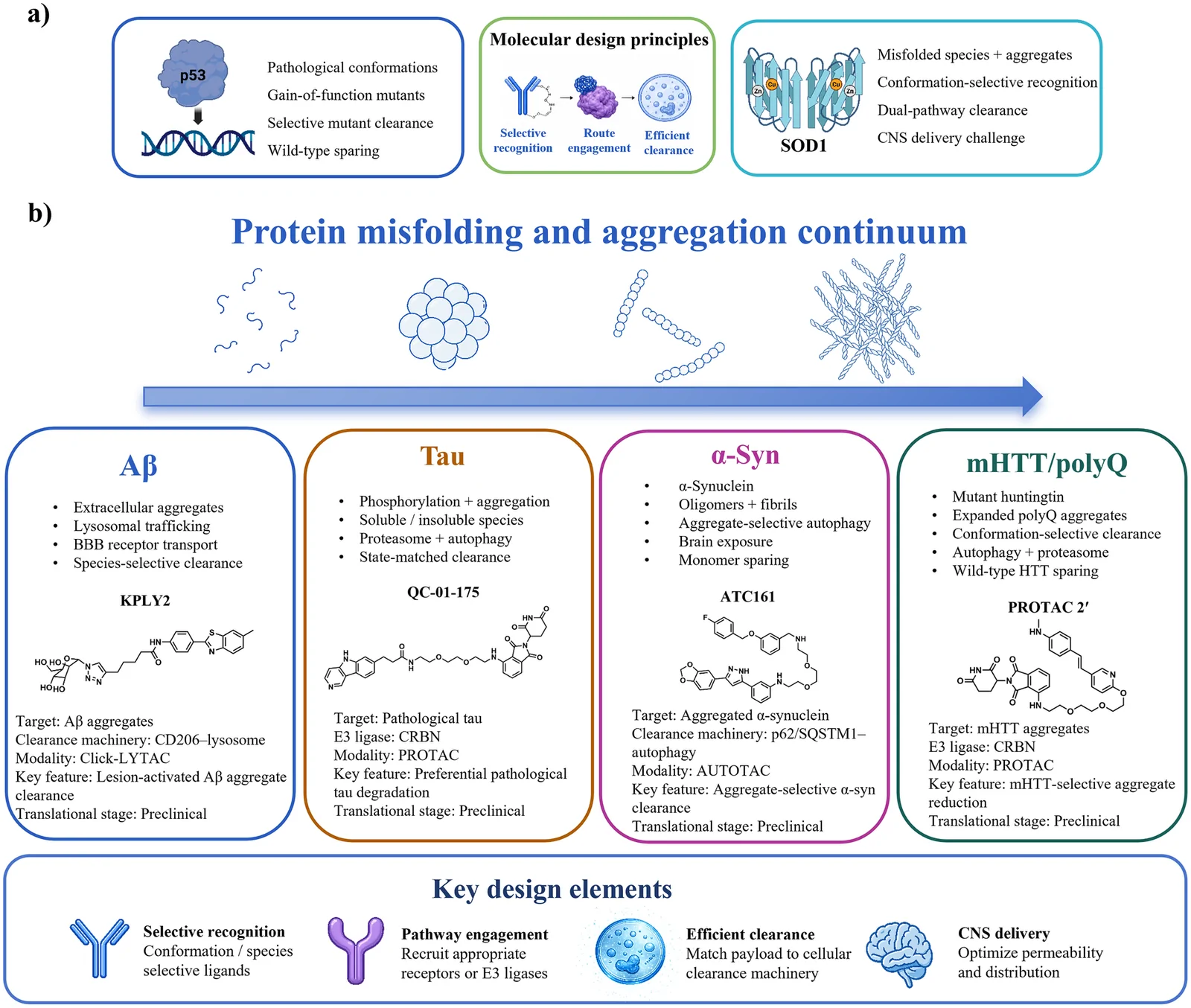
Pathological-state recognition and clearance-route selection for misfolded and aggregated proteins. **(a)** Misfolded p53 and SOD1 illustrate conformer-selective recognition and preservation of normal proteins. **(b)** Aβ, tau, α-synuclein, and mHTT illustrate the influence of aggregate scale and localization on clearance-route selection; representative molecules include KPLY2, QC-01-175, ATC161, and PROTAC 2′.

### 2.6 Subcellular mislocalization, aberrant cell-surface localization, and organelle damage

These abnormalities primarily involve protein mislocalization to inappropriate subcellular compartments, aberrant expression at the cell surface, or impaired organelle quality control (5). Because the same protein may perform essential physiological functions in its normal compartment, therapeutic strategies should target the mislocalized pool or damaged structure rather than indiscriminately reducing the protein throughout the cell (5). The following sections discuss targeted clearance of cytoplasmic TAR DNA-binding protein 43 (TDP-43), aberrant membrane proteins, and damaged mitochondria.

#### 2.6.1 TDP-43

TDP-43 is normally localized predominantly in the nucleus, where it regulates RNA splicing, transcript stability, and cryptic exon repression (252, 253). In amyotrophic lateral sclerosis and some forms of frontotemporal lobar degeneration, TDP-43 undergoes nuclear depletion, cytoplasmic mislocalization, aberrant phosphorylation, truncation, and aggregation (61, 62). Its pathology therefore combines loss of nuclear RNA-processing functions with a toxic gain of function in the cytoplasm (61, 62, 252). Nuclear depletion of TDP-43 can cause aberrant splicing of transcripts such as UNC13A, suggesting that indiscriminate reduction of total TDP-43 may further impair motor-neuron function (254). Although pathological TDP-43 also undergoes truncation, phosphorylation, and aggregation, it is classified primarily by spatial state because the therapeutic objective is to remove the cytoplasmic pathological pool while preserving the physiologically essential nuclear pool (61, 62).

Current direct-degradation studies have focused mainly on pathological cytoplasmic C-terminal fragments of TDP-43 (255). CRBN-recruiting PROTAC 2 uses an aggregate-binding moiety to recognize the TDP-43 fragment spanning residues 219–414 (255). In Neuro-2a cells, it reduced phosphorylated, high-molecular-weight, and detergent-insoluble C-terminal TDP-43, and these effects were attenuated by proteasome inhibition (255). The molecule also reduced C-terminal TDP-43 oligomers and, in a neuronal overexpression model in *Caenorhabditis elegans*, decreased aggregation and improved motor phenotypes. These findings provide proof of concept that proteasome-directed degraders can act on selected pathological TDP-43 species (255).

This study has clear limitations. The model relies on overexpression of a C-terminal fragment rather than endogenous full-length TDP-43 in patient-derived motor neurons (255). PROTAC 2 had little effect on endogenous full-length TDP-43, and the available evidence is insufficient to establish strict selectivity for pathological conformations or the cytoplasmic compartment (255). Moreover, a reduction in the insoluble fraction may reflect a shift in aggregation equilibrium after clearance of soluble precursors and does not demonstrate direct proteasomal processing of mature aggregates (255).

For TDP-43 degradation in amyotrophic lateral sclerosis or frontotemporal lobar degeneration, phosphorylated, truncated, or aggregated proteins in the cytoplasm should be selectively cleared while preserving the nuclear TDP-43 required for RNA processing (61, 62, 252, 254). Evaluation should distinguish nuclear from cytoplasmic, full-length from truncated, and soluble from insoluble protein pools, while concurrently assessing cryptic-exon events and motor-neuron function (252, 254, 255). Larger cytoplasmic aggregates may be mechanistically better suited to autophagy–lysosomal clearance, but small molecules validated in patient-derived neurons and the mammalian brain are not yet available (230, 255). Direct TPD evidence for fused in sarcoma (FUS) and other proteins with nucleocytoplasmic mislocalization is even more limited, and these remain targets requiring further validation (256). TDP-43 thus illustrates a form of selectivity that is primarily spatial rather than protein-wide: medicinal chemistry should aim to discriminate the pathological cytoplasmic pool from the essential nuclear pool, and compartment-specific pharmacodynamics may be more informative than total cellular D_max_ (61, 62, 252, 254, 255).

#### 2.6.2 Clearance of PD-L1 and overexpressed EGFR/HER2 at the cell surface

This section focuses on membrane proteins that can be recognized from the extracellular space; degradation of EGFR driven primarily by mutations in its intracellular kinase domain was discussed in Section 2.3.3. Programmed death-ligand 1 (PD-L1) promotes immune evasion by suppressing antitumor immunity (257). EGFR overexpression, amplification, or aberrant membrane trafficking can enhance receptor signaling (258), whereas amplification of human epidermal growth factor receptor 2 (HER2) occurs in subsets of breast and gastric cancers (32, 259). Clearance of the intact membrane protein may disrupt receptor dimerization, scaffolding functions, and membrane recycling in addition to blocking ligand binding or kinase activity (27, 36, 49, 258).

LYTACs simultaneously bind the extracellular domain of a target protein and a lysosomal trafficking receptor, directing membrane proteins through the endocytic–endosomal–lysosomal pathway (27, 49). First-generation LYTACs recruiting the cation-independent mannose-6-phosphate receptor (CI-M6PR) reduced cell-surface EGFR, establishing the feasibility of redirecting intact transmembrane receptors to lysosomes (27). LYTACs recruiting the asialoglycoprotein receptor (ASGPR) subsequently reduced EGFR and HER2 in hepatocyte contexts, showing that the tissue distribution of trafficking receptors can confer a degree of cell-type selectivity (49). Their applicability remains constrained by heterogeneity in ASGPR expression and tissue exposure (49).

PD-L1 degradation is intended primarily for cancer immunotherapy (257). Antibody-based PROTACs (AbTACs) use bispecific antibodies to recruit PD-L1 to the membrane-associated E3 ligase RNF43, promoting its internalization and lysosomal degradation (260). Covalent DNA aptamer LYTACs enhance PD-L1 clearance through aptamer-mediated recognition and covalent target occupancy and have shown immune activation and antitumor activity in mouse tumor models (261). MBL-LYTACs link a target-binding ligand to a lysosome-localizing group and promote the internalization and lysosomal accumulation of PD-L1, EGFR, PTK7, and folate receptor α, thereby reducing dependence on a single trafficking receptor such as CI-M6PR or ASGPR (262). Their pharmacological activity remains constrained by the internalization capacity, membrane recycling, and lysosomal retention of the target receptor (258, 262).

The value of membrane-protein degradation must be assessed in the context of the specific disease. For PD-L1, comparative studies should determine whether sustained checkpoint removal provides advantages over blocking antibodies in terms of resistance, tissue selectivity, and therapeutic durability (258, 260, 261). A reduction in cell-surface protein alone does not establish clinical superiority, and persistent clearance in normal tissues and immune cells may increase immune-related toxicity (258, 260, 261). For EGFR and HER2, lysosomal clearance is better suited to tumors driven by receptor overexpression, amplification, or aberrant membrane trafficking (27, 32, 49, 257, 259). When disease is driven by mutations in the intracellular kinase domain, mutation-selective PROTACs may better preserve the wild-type receptor (186, 187). Drug exposure in brain metastases, receptor resynthesis, and compensatory bypass signaling remain shared challenges for both strategies (35, 257). Current evidence is largely limited to cellular and animal studies and does not yet establish whether degradation of intact membrane receptors is superior to existing antibodies, antibody–drug conjugates, or kinase inhibitors (27, 49, 260–262). For cell-surface targets, receptor choice, internalization kinetics, recycling, and tissue distribution become design variables analogous to E3-ligase selection in proteasome-directed degraders; the optimal clearance route should therefore be matched to the disease mechanism, such as receptor overexpression vs. kinase-domain mutation (27, 49, 186, 187, 257, 260–262).

#### 2.6.3 Damaged mitochondria

Mitochondrial damage includes loss of membrane potential, respiratory-chain dysfunction, increased reactive oxygen species, mitochondrial DNA damage, and impaired mitochondrial dynamics (263). It therefore represents a defect in organelle quality control rather than a lesion involving a single protein (263, 264). Classical PROTACs can reduce specific mitochondrial proteins but cannot use the proteasome to remove an intact organelle containing a double membrane, mitochondrial DNA, and multiple protein complexes (23, 264). Clearance of whole mitochondria therefore relies primarily on selective mitochondrial autophagy (mitophagy) (23, 264).

AUTACs comprise a mitochondrial-targeting moiety and a guanine-derived autophagy tag, promoting K63-linked ubiquitination and selective autophagy (26). The mitochondria-targeting AUTAC4 enhanced clearance of fragmented and dysfunctional mitochondria and improved the mitochondrial network, membrane potential, and cellular energy state in fibroblasts derived from patients with Down syndrome (10). These findings suggest that, in diseases characterized by impaired mitochondrial quality control, controlled removal of damaged organelles may help restore mitochondrial homeostasis (26).

Mito-ATTECs link a mitochondria-targeting ligand to an LC3-binding moiety, recruiting mitochondria to autophagosomes for lysosomal clearance (265). Unlike AUTAC4, which emphasizes organelle quality control, sustained mito-ATTEC treatment reduced mitochondrial mass, mitochondrial DNA, and ATP levels, leading to mitochondrial depletion and bioenergetic collapse in selected tumor cells (265). This strategy exploits tumor-cell dependence on mitochondrial metabolism by driving persistent mitochondrial clearance to induce energetic failure and cell death (265).

The required extent of mitochondrial clearance differs across diseases. In neurodegenerative disorders, inherited mitochondrial diseases, and ischemic injury, dysfunctional mitochondria should be preferentially removed while sufficient healthy organelles are preserved to sustain energy metabolism (263, 264). Cancer therapy, by contrast, may exploit deeper and more sustained mitochondrial clearance to induce metabolic imbalance (265). Evaluation of mitochondrial degradation should therefore integrate TOM20 or mitochondrial dye signals with measurements of membrane potential, oxygen-consumption rate, ATP, reactive oxygen species, mitochondrial DNA, autophagic flux, and compensatory mitochondrial biogenesis (26, 265). A decline in mitochondrial markers alone should not be interpreted as evidence of functional benefit (26, 265).

AUTACs and mito-ATTECs remain largely at the stage of mechanistic validation in cellular and animal models, with no established evidence for human pharmacokinetics, tissue pharmacodynamics, or long-term safety (26, 265). Their clinical translation will depend on the ability to recognize disease-relevant states of mitochondrial damage while avoiding excessive depletion of healthy mitochondria in energy-demanding tissues such as neurons, cardiac muscle, and skeletal muscle (263–265).

For mislocalized proteins, membrane receptors, and damaged organelles, a common design requirement is to restrict clearance to the relevant compartment and tissue. The therapeutic window depends primarily on selectivity for the abnormal compartment, diseased tissue, or damaged structure, rather than on the depth of clearance across the entire cell (26, 264, 265). For organelle-directed clearance in particular, the dose-response relationship should be viewed as a quality-control window rather than a search for maximal clearance, because excessive removal can deplete functional organelles and create a mechanism-based toxicity distinct from incomplete target engagement (26, 265). Representative compartment-, cell-surface-, and organelle-directed clearance strategies discussed in this section are summarized in Figure 6.

**Figure 6:**
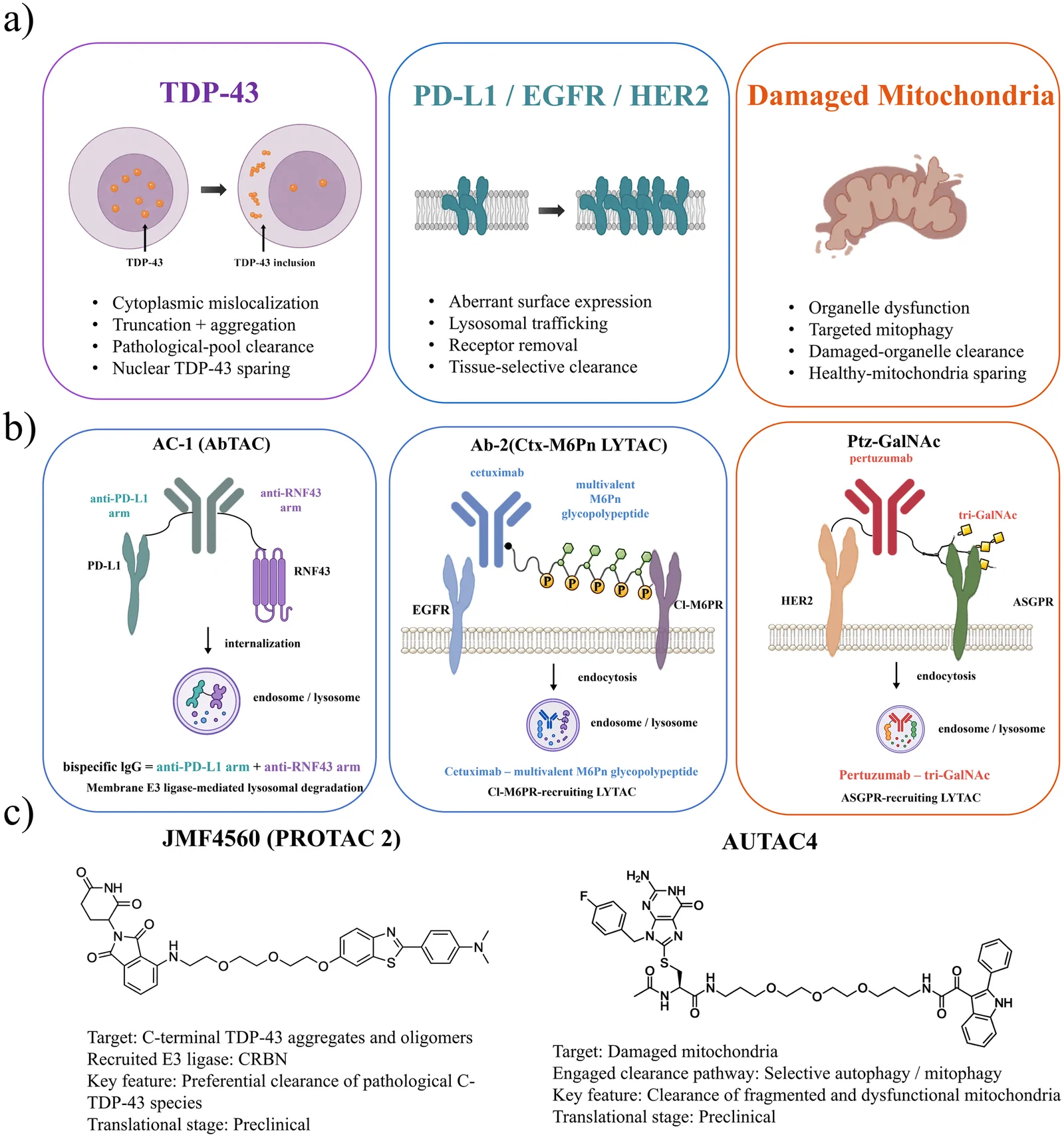
Representative targets and clearance strategies for protein mislocalization, aberrant cell-surface expression, and organelle damage. **(a)** Representative pathological spatial states, including cytoplasmic mislocalization of TDP-43, aberrant cell-surface expression of PD-L1/EGFR/HER2, and mitochondrial damage, highlighting the need for compartment- and tissue-selective clearance. **(b)** Representative clearance strategies, including AbTAC- and LYTAC-mediated lysosomal trafficking of cell-surface proteins and targeted mitophagy of damaged mitochondria. **(c)** Representative chemical degraders and autophagy-directed molecules developed for selective clearance of mislocalized proteins and damaged organelles.

## 3 Common challenges in the design, validation, and translation of targeted degradation and clearance for pathological protein states

Taken together, the representative cases discussed in Section 2 show that disease targets sharing a dominant pathological state can converge on similar medicinal-chemistry and pharmacological constraints despite substantial differences in protein identity and disease context (44, 56, 57). Targets defined by persistent protein dependence, activating alterations, or treatment resistance require adequate coverage of disease-relevant variants while preserving physiological protein function where necessary (54, 57, 187). Misfolded and aggregated species additionally require selective recognition of the pathological state and matching of substrate scale to an appropriate clearance pathway, whereas spatially abnormal proteins and damaged organelles demand tissue- or compartment-restricted clearance (230, 238, 255, 264, 265).

These biological requirements are translated into degrader performance through multiple, interdependent molecular design variables. For heterobifunctional degraders, these include the target-binding ligand and its binding mode, the attachment site or exit vector, linker length and conformational properties, the recruited E3 ligase, and the geometry and dynamics of the resulting ternary complex (45–47, 266). More broadly, degradation is further shaped by cellular permeability (267), intracellular unbound exposure (268), tissue distribution, target and clearance-machinery abundance, and target-protein resynthesis (57, 59, 268). Representative compounds for which these molecular design features can be related to degradation pharmacology and translational performance are summarized in Table 2.

**Table 2:** Representative degraders linking molecular design, degradation pharmacology, drug-like properties, and translational performance.

Compound	Target/pathological state	Chemical design	Mechanistic determinant/selectivity origin	Cellular degradation	Drug-like properties & exposure	*In vivo*/human PD	Medicinal chemistry lesson
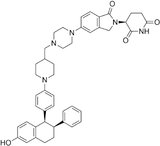 Vepdegestrant	ERα Persistent/mutant ER dependency	Oral heterobifunctional ER degrader	WT and mutant ERα degradation; clinical selectivity is primarily biomarker-driven	DC_50_ = 0.9 nM; D_max_ = 95%; 72 h; MCF7	Oral; sustained exposure	Tumor ER degradation up to 89% in patients	Oral drug-like optimization can translate PROTAC pharmacology into approved therapy.
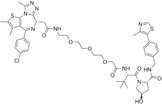 MZ1	BRD4/BET Transcriptional dependency	BET ligand–linker–VHL degrader	Folded cooperative ternary complex and *de novo* protein–protein interactions	DC_50_ ≈ 2.5 nM (pDC_50_ = 8.6); D_max_ = 100%; 24 h; HeLa	High efflux; poor oral exposure	*In vivo* PD not reported	Ternary-complex geometry can create selectivity beyond binary ligand binding.
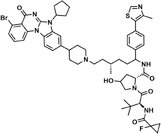 ACBI2	SMARCA2 SMARCA4-loss synthetic lethality	Bromodomain-directed SMARCA degrader; optimized architecture	Ternary-complex geometry and protein-surface differences drive SMARCA2 selectivity	DC_50_ = 1 ± 1 nM; D_max_ = 81 ± 7%; 18 h; RKO	Reduced efflux; oral F = 22%	Dose-dependent tumor SMARCA2 degradation in mice	Linker and ternary-complex optimization can enable paralog selectivity and oral exposure.
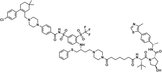 DT2216	BCL-XL Anti-apoptotic dependency	BCL-XL ligand–linker–VHL degrader	Low VHL abundance in platelets enables tissue-selective BCL-XL degradation	DC_50_ = 63 nM; D_max_ = 90.8%; 16 h; MOLT-4	Parenteral; no oral bioavailability	Rapid, sustained BCL-XL degradation in human leukocytes	E3-ligase expression can be exploited to achieve tissue-selective degradation.
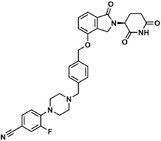 Mezigdomide	IKZF1/3 Myeloma transcriptional dependency	CRBN-binding CELMoD molecular glue	CRBN neosubstrate recruitment enhances IKZF1/3 degradation	Aiolos/Ikaros depletion = 80%–95% at 1 nM; 6 h; H929/MM1.S	Oral; linear exposure	Rapid, sustained Aiolos degradation in blood and tumor	Compact molecular glues can reprogram E3 substrate recognition without bifunctional architecture.
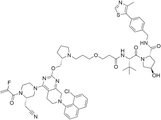 LC-2	KRAS G12C Oncogenic activating mutation	MRTX849-derived covalent G12C ligand–VHL degrader	G12C-specific covalent recognition enables mutant-selective KRAS degradation	DC_50_ = 0.59 ± 0.20 μM; D_max_ ≈ 80%; 24 h; NCI-H2030	Poor solubility; low bioavailability	*In vivo* PD not reported	Covalent mutant recognition can enable allele-selective degradation but restrict target coverage.
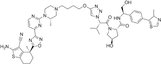 ACBI3	Oncogenic KRAS Multi-allelic activating mutations	VHL-recruiting KRAS degrader; linker-optimized	Ternary-complex and linker optimization broaden KRAS allele coverage	DC_50_ = 2 nM (G12D, GP5d)/7 nM (G12 V, SW620); 24 h	Parenteral; sustained exposure	Tumor KRAS^G12D^ reduced to 44% of control in mice	Ternary-complex optimization can broaden degradation across multiple oncogenic alleles.
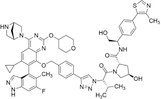 ASP3082 (Setidegrasib)	KRAS^G12D^ Oncogenic activating mutation	Noncovalent KRAS^G12D^ ligand–VHL degrader	Noncovalent G12D recognition with productive VHL ternary-complex formation	DC_50_ = 38 nM; 24 h; AsPC-1	i.v. QW; long t_1/2_	Median 88% KRAS^G12D^ degradation in human tumors	Productive degradation does not require covalent target engagement.
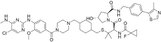 XL01126	LRRK2 Mutant multidomain signaling	VHL-recruiting LRRK2 degrader; optimized linker connectivity	Optimized linker connectivity and positive ternary-complex cooperativity	DC_50_ = 14 nM; D_max_ = 90%; 4 h; G2019S LRRK2 MEFs	Oral; brain exposure	*In vivo* target PD not reported	Degradation potency and CNS exposure can be co-optimized in bRo5 space.
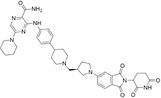 NX-2127	BTK Persistent/resistant BCR signaling	CRBN-recruiting BTK degrader with CELMoD activity	BTK degradation across resistant variants with additional CRBN-mediated IKZF1/3 degradation	BTK DC_50_ = 1.9 nM (WT)/1.9–9.7 nM (resistant variants); 24 h; TMD8	Oral QD; systemic exposure	>80% BTK degradation in patients; sustained with daily dosing	Degradation can overcome resistance while introducing useful or unwanted polypharmacology.

BCR, B-cell receptor; BET, bromodomain and extraterminal; bRo5, beyond the Rule of Five; CELMoD, cereblon E3 ligase modulator; CNS, central nervous system; CRBN, cereblon; DC_50_, half-maximal degradation concentration; D_max_, maximum degradation; F, oral bioavailability; i.v., intravenous; MEFs, mouse embryonic fibroblasts; PD, pharmacodynamics; PROTAC, proteolysis-targeting chimera; QD, once daily; QW, once weekly; t_1/2_, elimination half-life; VHL, von Hippel–Lindau; WT, wild type. pDC_50_ = −log10(DC_50_ [M]).

Rather than providing an exhaustive catalogue of degraders or ranking compounds solely according to DC_50_ or D_max_, Table 2 is intended to highlight transferable medicinal-chemistry principles. These include selectivity arising from induced ternary-complex geometry rather than binary ligand affinity alone (45, 47, 266), tissue selectivity generated by differential E3-ligase expression (147), the distinct consequences of covalent and noncovalent target recognition (163), the role of degrader architecture in mutation or paralog coverage (85, 112, 187, 266), and the need to balance cellular degradation potency with drug-like properties and pharmacologically effective tissue exposure (57, 108, 111, 267, 268). Because DC_50_ and D_max_ depend strongly on cellular context, treatment duration, assay format, and target abundance, quantitative values are compared only when derived under sufficiently similar experimental conditions (57, 59, 268). Building on these representative examples, the following sections examine the common molecular, pharmacological, and translational determinants that govern successful targeted degradation across pathological protein states (44, 56, 57).

### 3.1 Degradation efficiency, selectivity, and mechanistic validation

Mechanistic validation of TPD should cover the full sequence of events, including binding of the target protein to the clearance machinery, formation of the induced complex, productive ubiquitination or cargo recruitment, substrate trafficking, and final processing (27, 44, 230). Disruption at any step may limit protein reduction or cause the observed decrease to arise through a mechanism other than the intended degradation pathway (44, 59, 230). For proteasome-directed heterobifunctional degraders, ternary-complex cooperativity, geometry, and residence time can influence ubiquitination efficiency, although strong positive cooperativity is not an absolute requirement for degradation (45–47, 266). More important is whether the induced complex is sufficiently populated and adopts a productive conformation that presents suitable lysines for ubiquitination and an accessible region from which the proteasome can initiate unfolding and proteolysis (47, 266, 269, 270).

Productive ternary-complex formation is also concentration dependent. At low-to-intermediate concentrations, increasing heterobifunctional degrader exposure generally favors formation of the target–degrader–E3 complex, whereas excessive concentrations may preferentially populate binary target–degrader and E3–degrader complexes and thereby reduce productive ternary-complex formation, producing the so-called hook effect (45). The magnitude and concentration range of this effect depend on binary affinities, ternary-complex cooperativity and kinetics, target and E3 abundance, and intracellular free degrader concentration (45, 46, 268). Thus, stronger binary binding or higher compound exposure does not necessarily translate into deeper degradation, and DC_50_ and D_max_ should be interpreted together with the tested concentration range, treatment duration, and cellular context (45, 57, 59, 268). Importantly, not all degraders exhibit a pronounced hook effect within pharmacologically relevant concentration ranges, particularly when positive cooperativity or kinetic stabilization favors productive ternary-complex formation (45, 46).

Beyond degradation efficiency, assessment of degradation selectivity should integrate the binary ligand-binding profile, the properties of the induced complex, and the proteome-wide degradation profile (47, 266, 271). The attachment site, linker conformation, target-protein surface, and relative orientation of the target and E3 ligase can create a new protein–protein interface, causing the actual degradation profile to diverge from the binding profile of the parent ligand (47, 266, 271). Candidate evaluation should therefore proceed from target occupancy and complex formation to ubiquitination, protein reduction, and downstream functional effects, with quantitative proteomics used to identify unintended substrates (59, 271).

Different clearance modalities require tiered mechanistic evidence aligned with their respective pathways. Proteasome-directed degradation should be validated by demonstrating productive target–degrader–E3 complex formation, ligase dependence, ubiquitination, and proteasome dependence (44, 45, 59). Endocytic–lysosomal strategies should confirm trafficking-receptor binding, target internalization, endosomal transport, and lysosomal processing (27, 49). Autophagy-based strategies should assess cargo recognition, engagement of the intended autophagy machinery or adaptor (e.g., LC3 or p62), autophagic flux, and lysosomal dependence (230, 239). Studies should also include appropriate controls, such as inactive ligands, linkers, or recruitment modules, and exclude apparent protein loss caused by reduced transcription, translational inhibition, or cytotoxicity (59, 255).

For misfolded and aggregated proteins, reductions in total protein or the insoluble fraction may result from direct clearance, aggregate disassembly, suppressed production, depletion of soluble precursors, or a shift in the aggregation–disaggregation equilibrium (229, 230, 250, 255). Experiments should separately quantify soluble monomers, oligomers, fibrils, and insoluble species (229, 230, 238, 250, 255). These measurements should be combined with assays of seeding activity, conformation-specific species, pathological propagation capacity, and clearance-pathway blockade to determine whether the target pathological species is directly processed (229, 230, 238, 250, 255). Mechanistic validation should proceed in parallel with candidate screening rather than being deferred until after lead-compound selection as a supplementary explanation (45, 59, 271).

### 3.2 Molecular architecture and multidimensional SAR of heterobifunctional degraders

Medicinal-chemistry optimization of heterobifunctional degraders is intrinsically multidimensional because the target-binding ligand, attachment site, linker, E3-ligase ligand, and resulting ternary-complex architecture are strongly coupled (44, 47, 111, 266, 271). Consequently, SAR cannot be inferred from binary target affinity alone (47, 85, 111, 271). A modification that preserves or even improves target binding may reduce degradation by altering ternary-complex geometry, cooperativity, kinetics, or the cellular population of productive complexes (45–47, 111, 266). Conversely, relatively modest-affinity ligands can support potent degradation when they generate favorable induced protein–protein interactions (47, 85, 271). Degrader optimization should therefore treat cellular degradation, ternary-complex behavior, selectivity, and molecular properties as parallel rather than sequential objectives (44, 57, 111, 267, 268).

The target-binding ligand, or warhead, defines the initial binding mode but should not be optimized solely for maximal binary affinity (85, 271). Binding-site selectivity, residence time, mutant or conformational preference, and the availability of a synthetically accessible attachment position all influence degrader performance (46, 266, 271). The exit vector is particularly important because changing the linker attachment site can reorient the degrader relative to the target surface without substantially altering binary affinity (111, 266). Such changes may expose different target surfaces to the recruited E3 ligase, alter lysine accessibility, and generate distinct induced protein–protein interfaces (47, 111, 266). Exit-vector scanning should therefore be considered an independent SAR dimension rather than a late-stage synthetic convenience (111, 266). For covalent warheads, reactivity and reversibility must additionally be balanced against mutant selectivity, ternary-complex formation, degrader recycling, and off-target covalency (163).

The linker simultaneously controls ternary-complex geometry and whole-molecule properties (45–47, 108, 111, 266, 267). Linker length, composition, rigidity, branching, and points of attachment determine the accessible conformational ensemble and can alter cooperativity, complex residence time, degradation selectivity, permeability, solubility, and metabolic stability (46, 108, 111, 266, 267). Accordingly, systematic linker scanning should evaluate not only length but also topology and conformational restriction (108, 111, 266). Longer or more flexible linkers may broaden conformational sampling but can impose entropic penalties and worsen permeability, whereas rigid or semirigid linkers can preorganize favorable conformations when the underlying geometry is correctly defined (108, 111, 267). Linker optimization should therefore be coupled to measurements of both degradation and cellular exposure rather than interpreted from biochemical potency alone (57, 108, 267, 268).

The E3-ligase ligand is likewise an active SAR element rather than a passive recruitment handle (45, 47, 85, 111, 112). Ligand affinity, chemotype, attachment position, stereochemistry, and reversible or covalent engagement can alter E3 occupancy, ternary-complex geometry, cellular permeability, and resistance liability (45, 85, 111, 112, 163). Increasing E3-ligand affinity is not necessarily beneficial if it preferentially stabilizes nonproductive binary complexes or narrows the concentration range over which productive ternary complexes are populated (45, 85). E3-ligand optimization should therefore consider affinity, attachment vector, stereochemistry, engagement kinetics, and productive ternary-complex formation together with permeability and resistance liability (45, 85, 111, 112, 163).

Because these structural variables converge on the induced ternary complex, ternary-complex SAR should be incorporated directly into degrader optimization (45–47, 111, 266). Relevant parameters include ternary-complex stability, cooperativity, association and dissociation kinetics, structural or conformational complementarity, and the concentration range over which productive complexes are populated (45–47, 111). These parameters should be interpreted alongside cellular DC_50_, D_max_, degradation kinetics, hook-effect behavior, proteome-wide selectivity, and intracellular free compound concentration (45, 57, 59, 268). No single ternary-complex metric is universally predictive: highly cooperative complexes may fail to degrade if their geometry is nonproductive, whereas weakly cooperative or even negatively cooperative systems can remain efficient when they support appropriate ubiquitination geometry and kinetics (45–47, 85). The medicinal-chemistry objective is therefore not to maximize any individual binding parameter, but to identify a molecular architecture that balances productive ternary-complex formation, catalytic degradation, selectivity, permeability, and exposure (45–47, 57, 111, 267, 268).

Representative examples illustrate this multidimensional SAR. MZ1 demonstrated that a folded degrader conformation and *de novo* target–E3 protein interactions can generate degradation selectivity beyond the selectivity of the parent BET ligand (47). ACBI2 showed that selective SMARCA2 degradation can emerge from ternary-complex geometry despite the high homology of SMARCA2 and SMARCA4 (111). In KRAS degradation, linker and ternary-complex optimization contributed to the broad allele coverage achieved by ACBI3 (174), whereas optimization of linker connectivity and ternary-complex cooperativity in XL01126 was compatible with both potent LRRK2 degradation and brain exposure (201). These examples show that degrader SAR is best understood at the level of the complete molecular architecture rather than as independent optimization of two binding ligands and a spacer (47, 111, 174, 201).

### 3.3 Medicinal chemistry of molecular glues and neomorphic interfaces

Molecular glues achieve targeted degradation without the modular bifunctional architecture of PROTACs. Instead, a compact small molecule binds one component of an E3-ligase or target complex and creates or stabilizes a complementary protein surface that recruits a neosubstrate (129, 136, 139, 140). Consequently, molecular-glue activity is an interface-level pharmacology: binary affinity can contribute to complex population, but degradation efficiency and selectivity depend on the geometry, complementarity, and dynamics of the induced protein–protein interface (129, 136, 140). This principle is illustrated by CRBN-binding immunomodulatory drugs, which remodel the CRBN substrate-recognition surface to recruit IKZF1/3, and by CELMoDs that further optimize this ligand-shaped interface to enhance degradation (40, 41, 123, 124, 129).

The same concept extends beyond CRBN. Aryl sulfonamides such as indisulam and E7820 promote recruitment of RBM39 to DCAF15, with structural studies indicating that productive degradation depends strongly on complementarity between the DCAF15–ligand surface and the RBM39 RNA-recognition motif rather than on high-affinity binary binding alone (135, 136). Cyclin K glues provide a mechanistically distinct example: ligand binding to CDK12 generates a solvent-exposed molecular surface that promotes association of the CDK12–cyclin K complex with DDB1, and structure–activity studies show that kinase inhibition and cyclin K degradation can be tuned at least partly independently (139, 140). These systems demonstrate that apparently minor changes to solvent-exposed substituents can alter neosubstrate recruitment without proportionally changing binary target engagement (136, 140).

Medicinal-chemistry optimization of molecular glues should therefore treat the induced interface as the principal SAR unit (129, 136, 140). Relevant variables include the affinity and binding pose of the glue to its primary protein partner, the shape and polarity of solvent-exposed substituents, neosubstrate surface complementarity, complex cooperativity and kinetics where measurable, and the concentration range over which productive complexes are formed (129, 136, 140). Compact molecular-glue scaffolds may offer advantages in permeability and oral drug-like properties relative to large heterobifunctional degraders, but their discovery is less modular because the productive neomorphic interface is difficult to predict from the two proteins or the ligand in isolation (123, 124, 140, 267). Structural biology, chemoproteomics, quantitative proteomics, and systematic analogue screening are therefore particularly important for identifying productive interfaces and distinguishing intended neosubstrates from degradation polypharmacology (128, 129, 136, 140).

Selectivity and resistance should also be considered at the interface level. Changes in E3-ligase abundance or complex integrity, mutations in the ligase or neosubstrate surface, and altered expression of competing substrates can change degradation without abolishing binary ligand binding (126–129). Conversely, the capacity of a glue to recruit more than one neosubstrate may be therapeutically useful in selected contexts but can also create mechanism-based toxicity (40, 41, 128, 129). Accordingly, optimization should focus not on maximizing glue affinity *per se*, but on engineering a compact chemical surface that produces a productive, selective, and sufficiently durable neomorphic interface in the disease-relevant cellular context (129, 136, 140).

### 3.4 Molecular drug-likeness, cellular permeability, and effective exposure

Heterobifunctional degraders typically have high molecular weights, high polarity, and many rotatable bonds, placing them beyond conventional rule-of-five chemical space (267, 272). Some PROTACs can transiently reduce solvent-exposed polar surface area through intramolecular hydrogen bonding and folded conformations, thereby improving membrane permeability (272, 273). This behavior is jointly influenced by linker length, rigidity, and attachment orientation (108, 266, 273). The linker therefore not only determines the distance between the two ligands but also modulates the overall conformation, solubility, cellular uptake, and pharmacokinetic properties (108, 267, 273). As reflected by the orally active and CNS-exposed degraders included in Table 2, optimization of these properties must occur in parallel with degradation potency, because improvements in DC_50_ or D_max_ that are achieved at the expense of permeability or unbound tissue exposure may not translate into superior *in vivo* pharmacology (57, 111, 201, 268).

Sufficient unbound exposure in cells and diseased tissues is a prerequisite for subsequent target engagement and activation of the clearance mechanism (57, 268). Drug-likeness assessment should integrate passive permeability, transporter-mediated efflux, intracellular unbound drug concentration, target occupancy, degradation kinetics, metabolic stability, and tissue pharmacodynamics, rather than relying solely on logP, polar surface area, or total drug concentration in cell lysates (57, 267, 268). For targets in the CNS and solid tumors in particular, effective concentrations in brain tissue, the tumor interstitium, or intracellular compartments are more informative about pharmacological activity than total plasma exposure (57, 111, 201).

Permeability can be improved by rigidifying the linker, reducing the number of hydrogen-bond donors, adjusting the attachment site on the target-binding ligand, and controlling the overall molecular conformation (108, 266, 267, 273). Reversible covalent binding, prodrug design, and intracellular assembly may also reduce the need for sustained high exposure for selected targets (274–276). These strategies should be evaluated alongside unbound drug exposure and actual degradation activity, because improvements in physicochemical properties cannot substitute for validation of cellular pharmacological effects (57, 268, 274–276). Oral absorption, tissue distribution, metabolite identification, and pharmacokinetic–pharmacodynamic relationships should also be assessed early to reduce the risk that inadequate systemic exposure is recognized only after completion of *in vitro* optimization (57, 111, 201).

### 3.5 Selection and expansion of clearance machinery

CRBN and VHL benefit from well-established ligands and extensive structural characterization, but their tissue expression, subcellular distribution, and resistance mechanisms are not suited to every disease context (39, 44, 126, 127). Chemical proteomics and covalent-fragment screening have identified ligandable sites in E3 ligases such as DCAF16, RNF4, RNF114, and FEM1B, providing new recruitment modules that expand the degradable target space (277–280). The value of these E3 ligases extends beyond increasing the pool of available ligands, as their tissue expression and subcellular localization may confer selectivity (147, 149, 277). DCAF16, for example, is predominantly nuclear and may therefore be better suited to nuclear proteins, whereas other ligases may show distinct activity in specific tissues or under defined stress conditions (277).

Evaluation of new E3 ligases should extend beyond demonstrating degradation of a single target to assess ligase-complex integrity, E2 pairing, native substrates, tissue expression, subcellular localization, ligand occupancy, and long-term toxicity (39, 277–280). Covalent recruitment also requires evaluation of reactive-site selectivity, the effects of irreversible occupancy on physiological ligase function, and off-target reactivity (163, 277–280). For the same disease target, different E3 systems should be compared in terms of degradation profile, tissue pharmacodynamics, resistance mechanisms, and safety window, rather than solely by cellular DC_50_ or D_max_ (44, 57, 126, 127).

Direct proteasome recruitment can bypass E3 ligases and ubiquitin transfer. Linking a PSMD2-binding macrocyclic peptide to a target-binding ligand directed BRD4 to the 26S proteasome, providing proof of concept for E3-independent clearance (281). Small-molecule approaches that recruit the proteasomal cap are also being explored (282). Such molecules still require the target protein to contain an accessible disordered region from which the proteasome can initiate processing, and the recruitment ligand must not disrupt basal proteolysis (271, 272, 281, 282).

When multiple clearance pathways are feasible for the same target or pathological state, head-to-head comparisons should use similar or identical target-recognition elements. Selection of the clearance machinery should then be guided by the efficiency of pathological-species removal in disease-relevant cells, tissue pharmacodynamics, preservation of the normal protein, resistance mechanisms, and the safety window (44, 57, 126, 127, 277–282).

### 3.6 Spatial selectivity: tissue targeting, stimulus responsiveness, and subcellular localization

Once a candidate achieves adequate effective exposure, spatial selectivity further requires degradation activity to occur preferentially in diseased tissues, target cells, or pathological compartments (57). Tissue-targeting ligands, antibody or peptide conjugation, receptor-mediated uptake, and nanodelivery can increase exposure in diseased tissues, but their effectiveness depends on receptor expression, internalization, cargo release, and formulation stability (49, 283, 284). Evaluation should measure both exposure to the active degrader and target-protein reduction in diseased and normal tissues, rather than interpreting carrier accumulation as evidence of effective degradation (57, 283, 284).

External stimuli and disease-associated microenvironments can further restrict the site of degradation. PhotoPROTACs, PHOTACs, and photocaged molecules use light to control degrader activation, making them suitable for studies requiring high spatiotemporal resolution and for localized lesions (285–287). Their clinical application is limited by light penetration, phototoxicity, and reliance on specialized devices (285–287). Hypoxia-, reducing-environment-, or reactive-oxygen-species-responsive prodrugs, as well as bioorthogonal local assembly, can release or generate degraders at the disease site; however, heterogeneity in the triggering signal may lead to uneven degradation (75, 276, 284, 288, 289).

Such systems should report background activity in the unactivated state, fold activation, active-species kinetics, differences in tissue exposure, and safety, while providing evidence that non-target tissues are meaningfully protected (75, 283–289). Protein reduction after triggering demonstrates only that the system has been activated and is not sufficient to establish spatial selectivity.

Subcellular selectivity requires colocalization of the degrader, target protein, and clearance machinery within the pathological compartment (26, 265, 277). Nuclear proteins may be addressed using nuclear-localized E3 ligases or degraders with nuclear distribution, whereas mitochondrial proteins and intact mitochondria involve distinct requirements related to membrane permeability, mitochondrial quality control, and mitophagy (26, 265, 277). Increasing lipophilicity alone does not ensure localization to a specific organelle. Robust evidence should include organelle fractionation, live-cell imaging, controls lacking the localization moiety, compartment-specific target-occupancy measurements, and quantitative pharmacodynamics (265, 277). The design objective should shift from whole-cell protein reduction to effective clearance within the disease-relevant compartment (265, 277).

### 3.7 Incomplete degradation, durability of pharmacodynamic effects, and acquired resistance

Incomplete initial degradation may result from insufficient intracellular exposure to unbound drug, nonproductive complex formation, inaccessible ubiquitination sites or proteasome-initiation regions, protection of the target within protein complexes, inadequate E3 expression, or rapid target resynthesis (45, 57, 59, 267–270). Structural optimization can alter the attachment orientation of the target-binding ligand, linker conformation, and molecular valency (105, 266). Biological strategies include switching the recruited E3 ligase, changing the clearance system, or modifying the dosing schedule (57, 281, 290). Trivalent PROTACs can enhance complex stability through increased affinity and cooperativity, although their larger molecular size may impair permeability and pharmacokinetic properties (105, 267).

The duration of protein reduction is determined by drug exposure, degradation rate, and target-protein resynthesis (57, 59, 268). Candidates should be characterized using time-resolved pharmacokinetic–pharmacodynamic relationships to distinguish event-driven effects that persist after drug clearance from occupancy-driven effects that require sustained exposure (57, 59). For rapidly resynthesized targets, combination therapy may be designed around disease-relevant upstream regulation of transcription or translation; nonselective inhibition of protein synthesis is not suitable as a general solution (57, 59, 268).

Prolonged treatment pressure can also drive acquired resistance. Long-term exposure to BET degraders can select alterations in core components of the CRBN or VHL complexes (291). Genetic screens further show that different degraders share resistance genes associated with the ubiquitin system and drug efflux, while also exhibiting E3- and target-specific resistance mechanisms (292–294). In some models, switching to another E3 ligase, such as DCAF1, restored degradation in cells resistant to CRBN- or VHL-recruiting degraders, providing a mechanistic rationale for E3 switching (290). However, newly adopted ligase systems may themselves undergo further adaptive changes (290, 293).

Before candidates advance to late-stage preclinical development or clinical testing, they should undergo long-term drug-selection studies, genome-wide or focused CRISPR screens, target-protein mutational scanning, and validation with alternative E3 ligases (290–294). Samples collected after disease progression should also be analyzed for alterations in the target protein, E3 ligase complex, clearance pathway, drug efflux, and bypass signaling, rather than only for mutations in the ligand-binding site of the target (291–294). Rational selection of subsequent therapy requires distinguishing inadequate drug exposure, target escape, inactivation of the clearance machinery, and rewiring of disease pathways (57, 290–294).

### 3.8 Computationally assisted design, data standardization, and model validation

Computational methods can support conformational sampling of induced complexes, linker generation, analysis of accessible ubiquitination sites, and property prediction for molecules beyond conventional drug-like chemical space (267, 295–298). Ternary-complex modeling workflows, AIMLinker, PROTACable, and deep generative models have been used to narrow the candidate chemical space and guide linker design (295–298). These methods differ in their treatment of protein flexibility, solvent effects, and induced fit, and their accuracy varies substantially across target–E3 pairs (295, 297, 299). At present, scores derived from static complex models cannot reliably predict the depth or kinetics of cellular degradation or tissue pharmacodynamics (268, 295, 297, 299).

Computational methods and artificial intelligence are better suited to excluding clearly implausible structures, proposing synthetically accessible candidates, and prioritizing experiments than to replacing mechanistic or pharmacological validation (296–299). Generated structures must still undergo sequential evaluation of binary binding, complex formation, ubiquitination or cargo recruitment, quantitative proteomics, cellular degradation, and *in vivo* pharmacodynamics (44, 59, 271, 295–299). Model performance also requires prospective experimental validation, as results obtained solely within the training set or from random data splits do not adequately demonstrate extrapolative capacity (299).

Data heterogeneity is a major constraint on model generalizability (299, 300). Existing studies differ in their definitions of DC_50_ and D_max_, as well as in treatment duration, cell lines, assay methods, and controls for cell viability, while negative compounds and failed programs are rarely disclosed (57, 59, 299, 300). Datasets used for model training should, at a minimum, record target and E3 expression, compound structure, concentration–time relationships, degradation and recovery kinetics, experimental methods, cytotoxicity, proteome-wide selectivity, and pharmacokinetic–pharmacodynamic outcomes (57, 59, 268, 299, 300). Models intended for disease stratification should also incorporate tissue permeability, target-protein state, E3 or endocytic-receptor expression, and patient molecular characteristics, rather than directly extrapolating average patterns across cell lines to individual patients (57, 267, 299, 300).

### 3.9 Biomarkers, patient stratification, and clinical pharmacodynamics

Patient stratification for TPD requires not only confirmation that the relevant target protein is present, but also assessment of whether the diseased tissue provides an exploitable clearance environment (7, 23, 49, 57). Vepdegestrant uses ESR1 mutation status to define the population most likely to benefit, with patient selection supported by an FDA-authorized assay, whereas setidegrasib is being developed for KRAS G12D-mutant solid tumors (50–52, 176, 301). These examples show that clinical application depends first on disease reliance on a specific protein state rather than on average activity in an unselected population (50, 52).

Subsequent biomarkers should capture both the clearance machinery and drug-disposition processes, including the E3 ligase complex, endocytic receptors, autophagy–lysosomal function, drug efflux, target-protein resynthesis, and tissue exposure (45, 57, 59, 267–270). A single gene mutation may not be sufficient to predict the depth or duration of degradation (57, 59, 126, 127, 294).

Clinical pharmacodynamics should, whenever possible, directly measure target-protein reduction in diseased tissues and relate it to downstream pathways, disease phenotypes, and clinical outcomes (50, 52, 57, 59). Protein reduction in peripheral blood may reflect systemic exposure but does not necessarily indicate pharmacological activity in solid tumors, the brain, or fibrotic tissues (57). When repeated biopsies are impractical, molecular imaging, circulating tumor DNA, cerebrospinal fluid biomarkers, or downstream protein signatures may be used, but their quantitative relationship to tissue degradation must be established (51, 57, 301, 302).

Patient stratification should also account for target-protein dependence in normal tissues, the distribution of the clearance machinery, and physiological compensation after sustained degradation (7, 57, 147, 149). The clinical advantage of TPD cannot be established solely by deeper or more durable protein reduction; it must translate into improved efficacy in a defined molecular subgroup while limiting toxicity in normal tissues (50, 52, 147, 149). A biomarker-guided clinical translation framework integrating these variables is illustrated in Figure 7.

**Figure 7:**
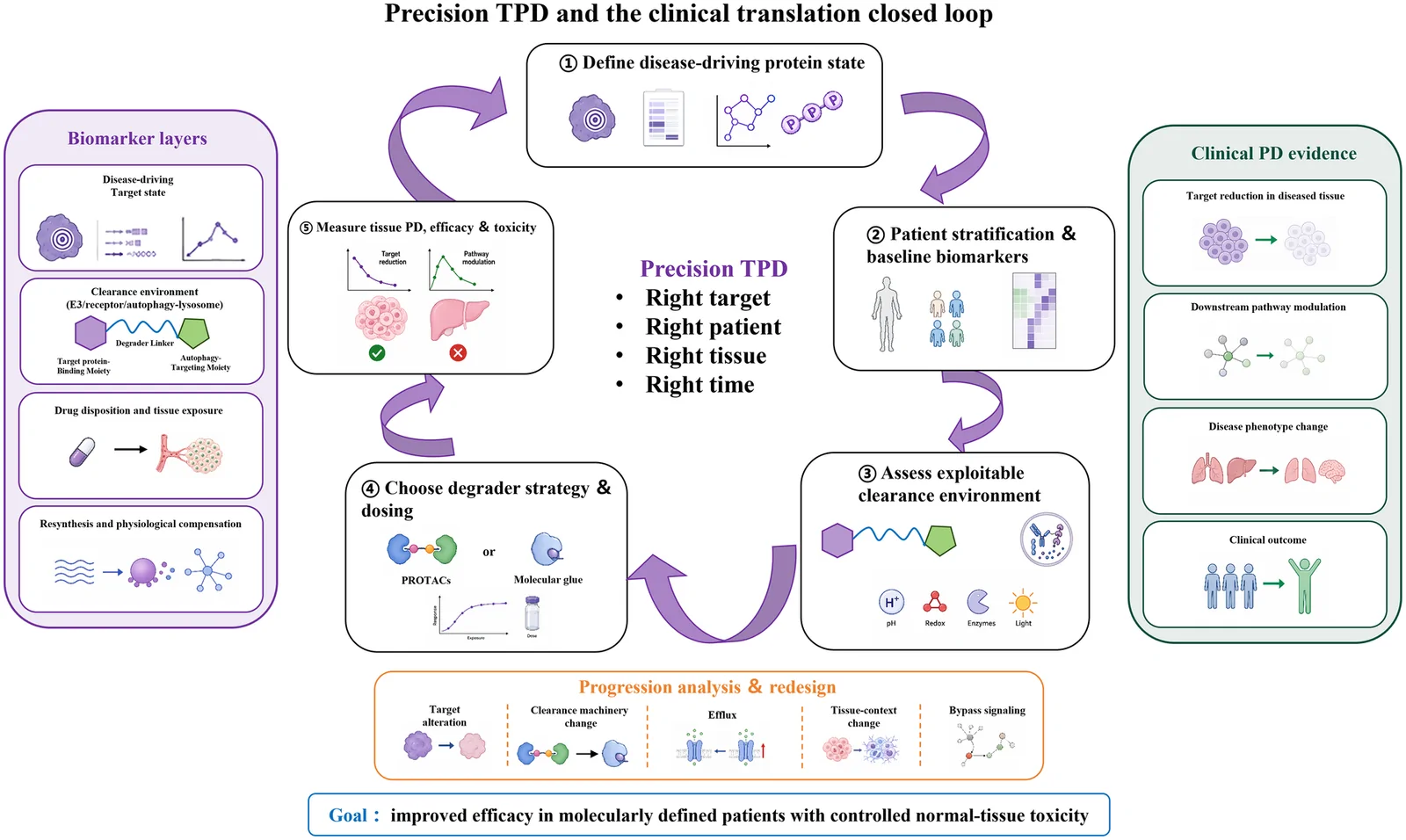
Biomarker-guided framework for patient stratification and clinical pharmacodynamics in targeted degradation and clearance. Predictive TPD integrates the disease-driving protein state, baseline biomarkers, and tissue-specific clearance capacity to guide degrader selection and dosing. Tissue pharmacodynamics, downstream pathway modulation, efficacy, and toxicity are then evaluated to establish target engagement and therapeutic response. Longitudinal analysis of target alterations, clearance machinery, efflux, tissue context, and bypass signaling enables iterative treatment redesign, with the ultimate goal of improving efficacy in molecularly defined patients while limiting normal-tissue toxicity.

### 3.10 Clinical translation gaps: safety, resistance, and tissue exposure

Clinical-stage TPD programs expose three recurrent translational constraints: safety, resistance, and effective tissue exposure (44, 56, 57). Toxicity may result from loss of physiological target function or unintended degradation. DT2216 demonstrates that tissue-selective E3 recruitment can widen the therapeutic window by eliminating on-target, off-tissue toxicity, particularly platelet toxicity resulting from unintended degradation in non-target tissues (147–152). Some CRBN-recruiting degraders introduce additional neosubstrate liabilities (40, 41, 128, 129), although the contribution of off-target degradation to clinical adverse events remains poorly defined (44, 56). Proteome-wide degradation profiles should therefore be interpreted together with human exposure and safety data (56–59).

TPD also changes, rather than eliminates, the resistance landscape. Degradation can overcome some inhibitor-resistant states when the mutation does not prevent productive degrader engagement, as illustrated by the activity of NX-2127 against several BTK variants (178–180). However, BTK A428D shows that degraders may inherit target-binding resistance when they use inhibitor-derived recognition scaffolds (183). Alternative recognition modes may similarly retain activity against selected EGFR C797S variants (184, 186, 187). In addition, degraders introduce resistance mechanisms involving the E3-ligase and ubiquitin machinery that are not shared by conventional inhibitors (126–128, 290–293). Resistance therefore depends on both target recognition and the integrity of the recruited degradation pathway (183, 290–293).

Clinical programs also expose the gap between systemic pharmacokinetics and tissue pharmacology (56, 57). HP518 showed less-than-dose-proportional exposure after repeated oral dosing (91), whereas intravenous setidegrasib achieved exposure-dependent KRAS G12D degradation in paired tumor biopsies (52). For CNS targets, ARV-102 illustrates the value of linking compartment-specific exposure with target reduction (201, 202). These examples reinforce the need for tissue- or compartment-specific human PK–PD relationships rather than reliance on plasma exposure or oral bioavailability alone (57).

TPD should therefore be compared with inhibition on clinically relevant endpoints rather than degradation depth alone (54–57). Key comparisons include tissue exposure, coverage of clinically relevant variants, effects on catalytic and non-catalytic target functions, resistance mechanisms, and normal-tissue toxicity (54–57). Direct clinical comparisons remain limited: vepdegestrant vs. fulvestrant provides one established example (50, 301), whereas randomized comparisons of BTK degraders with pirtobrutinib are still ongoing (181, 182). Mechanistic advantages of TPD should be distinguished from clinically demonstrated therapeutic advantages (54, 56).

## 4 Conclusions and perspectives

TPD and related targeted-clearance strategies have expanded from ubiquitin–proteasome-centered molecular glues and heterobifunctional degraders to endocytic–lysosomal, autophagy–lysosomal, and direct-proteasome-recruitment approaches (22, 27, 281). Translation is most advanced in oncology, where some agents have been approved or have entered late-stage clinical development. Immune-mediated inflammatory and CNS indications have yielded evidence of target-protein reduction in humans, whereas metabolic targets, misfolded and aggregated proteins, and damaged organelles remain largely at the preclinical stage (51, 53, 160, 193, 230, 265, 303).

This review uses the dominant pathological protein state, rather than the degradation platform or disease lineage, as the unit of analysis. By comparing similar pathological alterations across diseases and targets, it identifies shared clearance principles and translational bottlenecks. Current evidence supports selecting the clearance modality according to the principal disease-causing species. Persistent protein dependence, activating or resistance mutations, pathological conformations, aggregate scale, and abnormal spatial compartments impose distinct requirements for target recognition and clearance. The same protein may also require different clearance pathways depending on the pathological species being targeted. The “pathological protein state–clearance pathway–patient biomarker” framework can therefore connect target biology, molecular design, and clinical stratification, while providing transferable design principles and validation strategies for proteins with similar pathological states but limited degradation evidence. In this perspective, future targeted-clearance development may increasingly shift from technology-driven selection toward pathology-state-driven design, where identification of the dominant pathological species precedes the choice of recognition strategy, clearance machinery, and biomarker framework.

The next phase will require further harmonization of mechanistic and pharmacodynamic assessment standards, the establishment of quantitative relationships linking unbound drug exposure, degradation in diseased tissues, downstream functional changes, and clinical outcomes, and greater disclosure of negative compounds, failed programs, and conditions that do not produce degradation (57, 300). New E3 ligases, alternative clearance machinery, tissue targeting, and conditional activation may broaden the therapeutic window. Computational methods and artificial intelligence can support complex modeling and candidate prioritization, but their value will continue to depend on standardized data and prospective experimental validation (295, 300). The central challenge for the future development of TPD is to achieve predictable, quantifiable, durable, and selective clearance of the principal disease-causing species within defined patient subgroups and diseased tissues, while maintaining an acceptable safety window.
