# Supplementary material for: Disease-guided targeted protein degradation and clearance: matching strategies to pathological protein states
Source: Front Med (Lausanne). 2026 Oct 15;13:1967704. doi: 10.3389/fmed.2026.1967704 (PMC13717144; doi:10.3389/fmed.2026.1967704)
Supplement: Supplementary file 1 [file Supplementaryfile1.docx]

**Supporting Information**

**Figure S1. Chemical structures of representative targeted degraders and molecular glues directed against proteins characterized by elevated abundance or aberrant dependency.** a）ERα degraders; b）AR degraders; c）BET degraders; d）SMARCA2 degraders; e）IKZF1/3 molecular glues; f）RBM39 molecular glues; g）Cyclin K molecular glues; h）MCL-1 degraders; i）BCL-XL degraders; j）degraders targeting metabolic regulatory proteins. Chemical structures were redrawn by the authors based on structures reported in the primary literature.

**Figure S2. Chemical structures of representative degraders and selected parent or comparator ligands targeting persistent activation, oncogenic variants, and resistance mutations.** a）KRAS degraders; b）BTK degraders; c）EGFR degraders; d）IRAK/RIPK degraders; e）LRRK2 degraders. Chemical structures were redrawn by the authors based on structures reported in the primary literature.

**Figure S3. Chemical structures of representative agents targeting oligomeric, fibrillar, and aggregated proteins.** a) Aβ-targeting agents; b) Tau-targeting agents; c) α-synuclein-targeting agents; d) mutant huntingtin (mHTT)-targeting agents. Chemical structures were redrawn by the authors based on structures reported in the primary literature.

**Figure S4. Representative chemical structures and conjugates used for spatially selective and organelle-directed clearance.** a）TDP-43 degraders; b）AUTACs for damaged mitochondrial clearance; c）mito-ATTECs for mitochondria-directed autophagic clearance. Chemical structures were redrawn by the authors based on structures reported in the primary literature.
